# Grid cell firing patterns may arise from feedback interaction between intrinsic rebound spiking and transverse traveling waves with multiple heading angles

**DOI:** 10.3389/fnsys.2014.00201

**Published:** 2014-10-31

**Authors:** Michael E. Hasselmo, Christopher F. Shay

**Affiliations:** Department of Psychological and Brain Sciences, Center for Systems Neuroscience, Center for Memory and Brain, Graduate Program for Neuroscience, Boston UniversityBoston, MA, USA

**Keywords:** entorhinal cortex, stellate cells, whole-cell patch recording, spatial navigation, oscillatory interference

## Abstract

This article presents a model using cellular resonance and rebound properties to model grid cells in medial entorhinal cortex. The model simulates the intrinsic resonance properties of single layer II stellate cells with different frequencies due to the hyperpolarization activated cation current (h current). The stellate cells generate rebound spikes after a delay interval that differs for neurons with different resonance frequency. Stellate cells drive inhibitory interneurons to cause rebound from inhibition in an alternate set of stellate cells that drive interneurons to activate the first set of cells. This allows maintenance of activity with cycle skipping of the spiking of cells that matches recent physiological data on theta cycle skipping. The rebound spiking interacts with subthreshold oscillatory input to stellate cells or interneurons regulated by medial septal input and defined relative to the spatial location coded by neurons. The timing of rebound determines whether the network maintains the activity for the same location or shifts to phases of activity representing a different location. Simulations show that spatial firing patterns similar to grid cells can be generated with a range of different resonance frequencies, indicating how grid cells could be generated with low frequencies present in bats and in mice with knockout of the HCN1 subunit of the h current.

## Introduction

An understanding of cortical function requires that network dynamics be linked to intrinsic cellular properties. The model presented here links cellular properties of resonance in neurons in the medial entorhinal cortex (MEC) to the properties of grid cells. Grid cells exhibit spiking activity as a rat forages in a regular array of locations that fall on the vertices of tightly packed equilateral triangles (Fyhn et al., 2004; Hafting et al., 2005; Moser and Moser, 2008). The model presented here links the difference in size and spacing of grid cell firing fields along the dorsal to ventral axis of MEC (Hafting et al., 2005; Stensola et al., 2012) to the intrinsic resonance properties of stellate cells in layer II of MEC along this axis (Giocomo et al., 2007; Giocomo and Hasselmo, 2009; Boehlen et al., 2010; Heys et al., 2010; Canto and Witter, 2012; Pastoll et al., 2012a; Shay et al., 2012). These resonance properties depend upon the hyperpolarization activated cation current (h current) (Dickson et al., 2000; Haas and White, 2002; Erchova et al., 2004; Fransén et al., 2004; Rotstein, 2014) which also causes rebound spikes after hyperpolarization (Alonso and Llinas, 1989; Alonso and Klink, 1993). The model also addresses differences in resonance frequencies in layer II neurons from slices of MEC in the bat and the rat (Heys et al., 2013) and from mice with knockout of the HCN1 subunit of the h current (Giocomo and Hasselmo, 2009).

The models presented here build on elements of previous grid cell models including oscillations and attractor dynamics. Models of grid cells using sustained, fixed-point attractor dynamics (Fuhs and Touretzky, 2006; McNaughton et al., 2006; Guanella et al., 2007; Burak and Fiete, 2009; Milford et al., 2010; Cheng and Frank, 2011; Pastoll et al., 2012b; Bush and Burgess, 2014) generate many features of grid cells including the shared orientation and spacing of grid cells within a module (Hafting et al., 2005; Barry et al., 2007; Stensola et al., 2012). The early attractor models (Fuhs and Touretzky, 2006; McNaughton et al., 2006; Guanella et al., 2007; Burak and Fiete, 2009) did not address maintaining the attractor across cycles of the low frequency theta-rhythmic firing observed in entorhinal cortex neurons (Jeffery et al., 1995; Hafting et al., 2008; Brandon et al., 2011, 2013; De Almeida et al., 2012), but this has been addressed in more recent models (Navratilova et al., 2012; Pastoll et al., 2012b; Bush and Burgess, 2014).

In one recent attractor model (Navratilova et al., 2012), intrinsic rebound spiking allowed reactivation of the attractor state on each theta cycle to generate spatial periodicity, and replicate the phase precession of grid cell spiking relative to theta rhythm oscillation. Earlier models used this mechanism to simulate short-term memory (Lisman and Idiart, 1995) and theta phase precession (Jensen and Lisman, 1996). These models resemble the model presented here, but they used a depolarizing rebound based on spikes in an integrate-and-fire model (Navratilova et al., 2012). This previous dependence on spiking prevented explicit simulation of subthreshold properties caused by h current such as resonance and rebound from inhibition studied here, and the membrane potential oscillations seen in intracellular recordings of grid cells (Domnisoru et al., 2013; Schmidt-Hieber and Hausser, 2013). That previous paper also did not use an interaction with phases of network oscillations.

The model presented here builds on the oscillatory interference models of grid cells developed by Neil Burgess (Burgess et al., 2007; Burgess, 2008; Bush and Burgess, 2014) and variants of this model (Blair et al., 2007, 2008; Hasselmo, 2008; Zilli and Hasselmo, 2010; Welday et al., 2011). Oscillatory interference models (Burgess, 2008) are effective in simulating the theta phase precession of grid cells shown in one dimensional tracks (Hafting et al., 2008; Eggink et al., 2014) and two-dimensional environments (Burgess, 2008; Climer et al., 2013), building on the models of theta phase precession in hippocampal place cells (O'Keefe and Recce, 1993; O'Keefe and Burgess, 2005). In theta phase precession, grid cells start out firing at late phases of theta cycles when a rat enters the firing field of the cell and gradually shift to earlier phases of firing as the rat exits the firing field.

Oscillatory interference models could address the correlation between the size and spacing of grid cell firing fields at different dorsal to ventral anatomical positions (Hafting et al., 2005; Sargolini et al., 2006; Stensola et al., 2012) with the intrinsic spiking frequency measured with autocorrelograms of extracellular spiking in awake, behaving rats (Jeewajee et al., 2008; Stensola et al., 2012) and the intrinsic resonance frequencies measured with intracellular recording of membrane potential in slices (Giocomo et al., 2007; Hasselmo et al., 2007; Giocomo and Hasselmo, 2008a). Changes in intrinsic properties due to knockout of the HCN1 subunit of the h current channel alters the spacing and size of grid cell firing fields (Giocomo et al., 2011). However, previous models do not directly address how intrinsic resonance can influence grid cell firing. Early oscillatory interference models suggested that membrane potential oscillations could interact within single neurons (Burgess et al., 2007; Hasselmo et al., 2007), but later analysis showed that subthreshold oscillations of different phase cannot coexist within a single neuron (Remme et al., 2009, 2010) and do not change linearly with depolarization (Yoshida et al., 2011). Subsequent models showed oscillatory interference dependent on network interactions (Blair et al., 2008; Burgess, 2008; Hasselmo, 2008; Zilli and Hasselmo, 2010; Welday et al., 2011; Bush and Burgess, 2014). A related model used waves spreading across the cortical surface for coding location by firing phase and resulted in decoding activity with hexagonal interference patterns (Nadasdy, 2009, 2010). However, these models have not explicitly simulated how intrinsic rebound spiking could interact with network mechanisms to influence the size and spacing of grid cell firing fields. The model presented here focuses on the role of intrinsic resonance and rebound in causing phase shifts in spiking relative to network oscillations. This work extends beyond a previous model in which rebound spiking was used to simulate firing fields and theta phase precession along a one-dimensional trajectory (Hasselmo, 2013). This paper compares rebound to excitatory and inhibitory synaptic input, extends simulations to two dimensional trajectories, and addresses interactions of traveling waves with multiple heading angles.

## Methods

There are five groups of different simulations presented here. The first group in **Figures 4–7** use a simple model of resonance to generate spatially periodic activity using feedback excitation of a single population of stellate cells via conjunctive cells. The second group in **Figures 8**, **9** use the simple resonance model with conjunctive cells gating feedback inhibition between two populations of stellate cells. The third group in **Figures 10**, **11** use a spiking model of stellate cells with conjunctive cells gating feedback inhibition to show one dimensional propagation of activity. The fourth group in **Figures 12**, **13** use the spiking model of stellate cells with conjunctive cells gating feedback inhibition to show two-dimensional propagation of activity regulated by traveling waves to conjunctive cells in four different directions. Finally, the fifth group in **Figures 14**, **15** focus on a simplified feedback interaction of grid cells with multiple traveling wave inputs with different directions to show how feedback interactions can select a smaller number of traveling wave directions as the dominant input.

### Link to resonant properties

**Figure 1:**
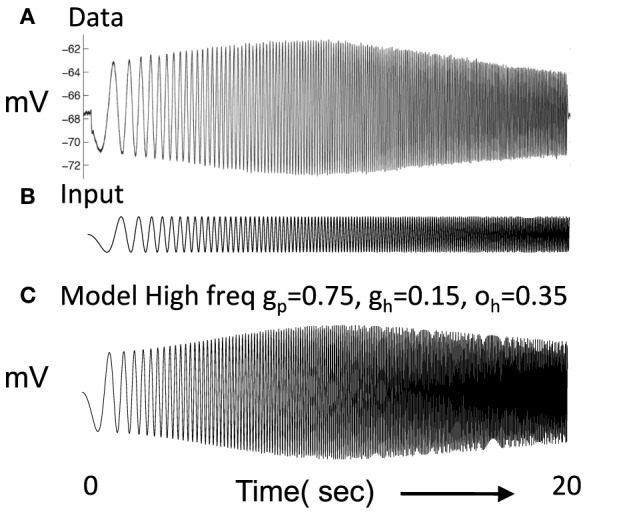
**(A)** Experimental data shows the response of a layer II stellate cell in MEC neuron to a chirp function (Shay et al., 2012). In response to oscillating current injection that increases in frequency, the neuron shows a gradual increase in amplitude of membrane potential oscillation that reaches a peak at resonance frequency and then decreases. **(B)** The chirp function input sweeps from 0 to 20 Hz over a period of 20 s. **(C)** The resonance frequency of the modeled stellate cell Equation (1) can be seen in the response to the chirp function. In this simulation, *g*_*p*_ = 0.75, *g*_*h*_ = 0.15, *m* = 1, and *o*_*h*_ = 0.35, give resonance frequency of 10.2 Hz.

A major focus was the simulation of resonance properties of single medial entorhinal neurons in response to current injection sweeping through increasing frequencies (Haas and White, 2002; Erchova et al., 2004; Fransén et al., 2004; Giocomo et al., 2007; Canto and Witter, 2012; Shay et al., 2012), as shown in Figure 1A. The current injection sweeping through frequencies was generated by the MATLAB chirp function and used to measure the impedance amplitude profile (ZAP). The resonance properties arise from intrinsic currents including the hyperpolarization activated cation current (h current) as described in layer II stellate cells of the MEC (Dickson et al., 2000). Previous models addressed these resonance properties (Dickson et al., 2000; Erchova et al., 2004; Fransén et al., 2004; Schreiber et al., 2004; Izhikevich, 2007; Engel et al., 2008; Rotstein, 2014; Rotstein and Nadim, 2014). The h current has a slow time course such that it responds to the slow hyperpolarizing phase during lower frequencies at the start of the chirp function with a more rapid depolarization that occurs at earlier phases relative to input and decreases membrane potential changes. Later when the frequencies in the chirp function match the time constant of the h current, the h current adds to the oscillation amplitude, causing a peak in the amplitude of membrane potential response. At higher frequencies of the chirp function, the h current response to hyperpolarization lags behind the input causing a decreased change in membrane potential.

Here the resonance properties of entorhinal neurons are modeled with linear coupled differential equations with oscillatory dynamics (Hasselmo, 2013). This differs from many previous oscillatory interference models that used sinusoids to represent oscillations (Burgess et al., 2005, 2007; Blair et al., 2007, 2008; Hasselmo et al., 2007; Burgess, 2008; Hasselmo, 2008; Hasselmo and Brandon, 2008). The sinusoids in those models could represent frequency and phase of oscillations but kept amplitude constant. Coupled differential equations allow the simulation of resonance frequency and strength in single neurons, as well as the change in response amplitude with circuit interactions.

### Resonant neurons

The equations of this simple model of resonance represent the change in membrane potential of an individual neuron *v* relative to resting potential (zero in these equations), and the change in activation *h* of the hyperpolarization activated cation current as follows:

(1) $$dv/dt\;=\;-g_{p}v+g_{h}h+I_{in}$$

(2) $$dh/dt\;=\;-mv-o_{h}h$$

The membrane potential *v* has passive decay modeled by the parameter *g*_*p*_, for decay back toward resting potential. The h current is a cation current that depolarizes the cell, so when h current is positive, it depolarizes the cell proportional to the parameter *g*_*h*_. The h current *h* is turned off by depolarization, so when *v* goes to positive values, it decreases the magnitude of *h* in proportion to *mv*. The h current is activated by hyperpolarization, so when *v* goes to negative values, it increases the magnitude of h in proportion to *mv*. The h current decays according to parameter *o*_*h*_ which was set to either 0.35 or 0.1.

The mathematical properties of these equations are well described (pp. 89–97 of Hirsch and Smale, 1974; Rotstein, 2014; Rotstein and Nadim, 2014, pp. 101–106 of Izhikevich, 2007). Here, parameters were chosen to give properties of resonance frequency that resemble the experimental data using the ZAP protocol. The dynamics of the network described below depend upon the resonance frequency of simulated stellate cells relative to the frequency of medial septal input *f* described below. The equations above can be algebraically reduced to the characteristic equation for a damped oscillator with forcing current:

(3) $$d^{2}v/dt^{2}+(g_{p}+o_{h})dv/dt+(g_{h}m+g_{p}o_{h})v=(1+o_{h})I_{in}$$

This second order version of the equation could also be used to create a wave equation for transverse waves defined relative to the spatial location coded in the environment. The undamped resonance frequency of this equation is $f=\sqrt{\left(g_{h}m\;+\;g_{p}o_{h}\right)}/2\pi$. For example, with a time step of 0.01 s, the parameters *g*_*p*_ = −0.49, *g*_*h*_ = 0.24, *m* = −1, *o*_*h*_ = −0.35 give *f* = 10.2 Hz. These parameters work well in **Figures 3–7**. However, the network dynamics also depend upon the strength of synaptic interactions, so the quantitative network dynamics cannot be determined only by Equations (1) and (2). Equations were solved in MATLAB using simple forward Euler methods, and qualitatively similar results were obtained using the ode45 solver (Runge-Kutta) in MATLAB.

The parameters were chosen to replicate resonance properties of stellate cells in layer II of MEC as shown in Figure 1A (Shay et al., 2012) in response to current injection consisting of the chirp function in Figure 1B, in which the frequency of the input current changes linearly from zero Hertz to 20 Hertz over 20 s. These functions are sometimes referred to as ZAP currents, where ZAP refers to the impedance amplitude profile computed in response to the chirp. In Figure 1C, a simulated neuron using the above equations shows a gradual increase in amplitude of oscillatory response to current injection until it reaches a peak response at the resonant frequency after which the amplitude of the oscillatory response decreases. This resembles the resonance response in the recording from a layer II stellate cell. The plot shown in Figure 1C used *g*_*p*_ = −0.75, *g*_*h*_ = 0.15, *m* = −1, *o*_*h*_ = −0.35 give *f* = 10.2 Hz. However, it was more difficult to balance the network dynamics with *g*_*p*_ = −0.75, so some network simulations used a lower value of *g*_*p*_.

**Figure 2:**
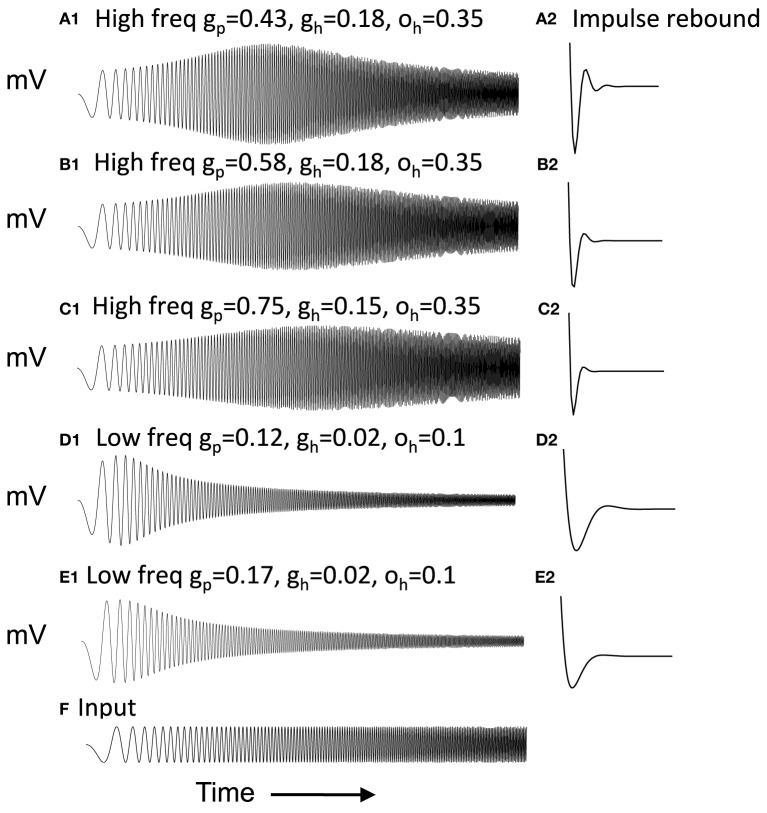
**Examples of neuron responses showing resonance at different frequencies that allow effective network function (A,B,D) except when *g*_*p*_ is too large (C,E).** Column 1 Responses of neurons to the chirp stimulus with different properties of resonance and damping in each row. **(A1)** Resonance using parameters of *g*_*p*_ = 0.43, *g*_*h*_ = 0.18 and *o*_*h*_ = 0.35. **(B1)** Resonance with *g*_*p*_ = 0.58, *g*_*h*_ = 0.18, and *o*_*h*_ = 0.35. **(C1)** Resonance using *g*_*p*_ = 0.75, *g*_*h*_ = 0.15, and *o*_*h*_ = 0.35. **(D1)** Resonance with *g*_*p*_ = 0.11, *g*_*h*_ = 0.01, and *o*_*h*_ = 0.1. **(E1)** Resonance with *g*_*p*_ = 0.16, *g*_*h*_ = 0.01, and *o*_*h*_ = 0.1. Column 2 With depolarized initial conditions, the neurons show a rebound depolarization with low *g*_*h*_ **(A2,B2,D2)** similar to data (Navratilova et al., 2012). This rebound depolarization is weak in **(C2,E2)**. **(F)** Input current for **(A1–E1)** consists of chirp stimulus sweeping through different frequencies.

Figure 2 Column 1 shows how the model can replicate different resonance properties in experimental data with different values of parameters *g*_*h*_ generating high (Figures 2A–C) and low resonance frequencies (Figures 2D,E). Examples 2C and 2E have the lowest resonance strength. The network model with excitatory connections below works best with the parameters shown in Figure 2A, but still works effectively with parameters shown in Figures 2B–D. The model with inhibitory connections works better across the full range of parameters.

Figure 2 Column 2 shows that with depolarized initial conditions, neurons show rebound depolarization that could activate the next oscillation cycle of an attractor. These traces in column 2 resemble the sequence of afterhyperpolarization and afterdepolarization that occurs after spikes in the experimental data from intracellular recording from stellate cells (Giocomo et al., 2007; Giocomo and Hasselmo, 2008b; Navratilova et al., 2012). The time course differs along the dorsal to ventral axis of MEC, with a shorter recovery time constant of afterhyperpolarization resulting in faster afterdepolarization in stellate cells from more dorsal slices (Navratilova et al., 2012). Network attractor dynamics work well when rebound depolarization is strong, as shown in Figure 2A2 and is still effective with one cycle of rebound as shown in Figures 2B2,D2. Weaker resonance strength corresponds to weaker rebound depolarizations in these neurons. **Figures 8–13** later show that network dynamics are easier to maintain with rebound due to hyperpolarizing inhibitory input rather than the depolarizing input shown here.

Another important property associated with resonance is the phase shift of the membrane potential in response to a sinusoidal current injection. When resonance frequency is higher than the input frequency, the simulated stellate cells show a phase difference in which the oscillations lead each cycle of the sinusoidal input. This difference in phase of the rebound depolarization can shift the phase of stellate cells relative to input from the medial septum to the network as described below, allowing a progressive shift in oscillatory activity within the network.

#### Network interactions of stellate cells

**Figure 3:**
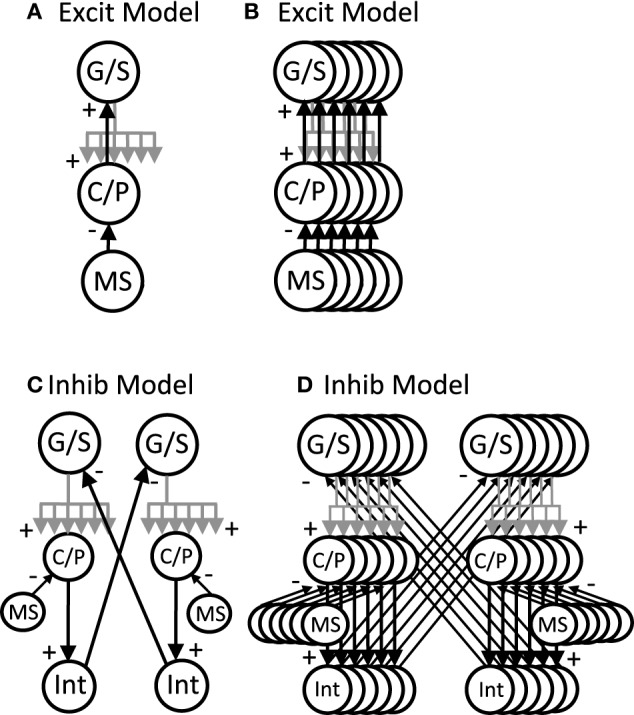
**Overview of circuit connectivity of both the excitatory and inhibitory model. (A)** Basic circuit diagram of Excitatory Model. Each grid/stellate cell (G/S) sends excitatory connections to the full population of conjunctive/pyramidal cells (C/P). Each conjunctive pyramidal cell gets oscillatory input from a medial septum neuron (MS) with a different phase. **(B)** Excitatory model diagram showing a full population of six G/S cells, and six C/P cells receiving six different MS inputs with different phase. This network is used in Figures 4–**7**. **(C)** Inhibitory model diagram showing that a G/S cell sends output that converges with MS input to a conjunctive cell C/P. The activity of the conjunctive cell C/P activates an interneuron that contacts a separate G/S cell that spikes in alternating theta cycles. **(D)** In the full inhibitory feedback model, each G/S cell projects to a full line of conjunctive cells receiving MS input. The convergence of G/S and MS input to conjunctive cells activates individual cells that activate individual interneurons. The interneurons projects to a separate population of G/S cells, allowing simulation of theta cycle skipping in the model in **Figures 8–13**.

The network simulations presented here generate patterns of activity based on network interactions summarized in Figure 3. The stellate cells in the model are proposed to be grid cells, so they are labeled with G/S in the figures and their membrane potential is designated by *v*_*g*_. The network dynamics involve an interaction of the resonant properties of stellate cells with the activity of other neurons receiving oscillatory input at different phases that are directly or indirectly regulated by rhythmic input from the medial septum. The medial septum has been shown to regulate theta rhythm oscillations in the entorhinal cortex (Mitchell et al., 1982; Jeffery et al., 1995; Brandon et al., 2011) and contains neurons that spike coherently with theta rhythm oscillations at a wide range of phases (Bland, 1986; King et al., 1998). As noted above, the network interactions of stellate/grid cells are analyzed in two different versions of the network model: (1) The excitatory model, and (2) The inhibitory model.

#### Excitatory model

**Figure 4:**
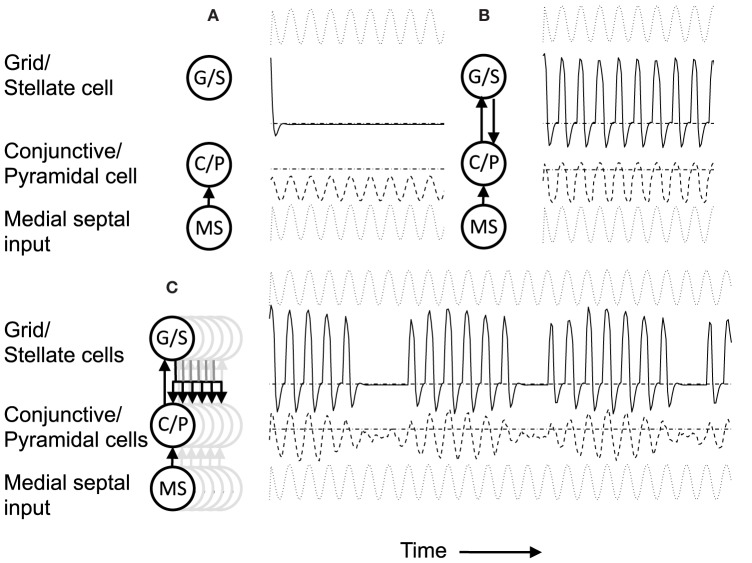
**(A)** Building network activity. **(A)** Single grid/stellate cell (G/S) with depolarized initial conditions shows rebound depolarization of membrane potential *v*_*g*_ before settling to zero. The conjunctive/pyramidal cell (C/P) shows subthreshold sinusoid oscillations of membrane potential *v*_*i*_ in response to sinusoidal input from medial septum (MS). **(B)** Addition of bidirectional excitatory synaptic connections allows maintenance of oscillatory activity. The grid cell potential *v*_*s*_ starts out above threshold (dash-dot line). Therefore, it depolarizes the membrane potential *v*_*i*_ of the conjunctive/pyramidal cell and the C/P cell crosses threshold (dashed line indicates level where *v*_*i*_ > θ_*i*_) and sends output to the corresponding G/S cell. Due to rebound depolarization in the G/S cell, on the next cycle, the potential *v*_*s*_ crosses threshold and gives excitatory input to the C/P cell *v*_*i*_ that then crosses threshold and sends feedback to *v*_*s*_. Thus, the single units *G/S* and *C/P* maintain oscillatory activity based on rebound depolarization. **(C)** Within a network, a single grid/stellate cell (G/S) with nonzero initial conditions shows rebound depolarization that is fast relative to the frequency of sinusoidal oscillations from MS input to the conjunctive/pyramidal cell (C/P). The higher resonance frequency causes the rebound depolarization of the G/S cell to match the earlier phase of a different C/P cell that gives feedback to a different stellate cell, causing a gradual decrease in the amplitude of the oscillations in the C/P cell and G/S cell shown here. The activity shifts between coupled pairs of cells with earlier and earlier phase.

**Figure 5:**
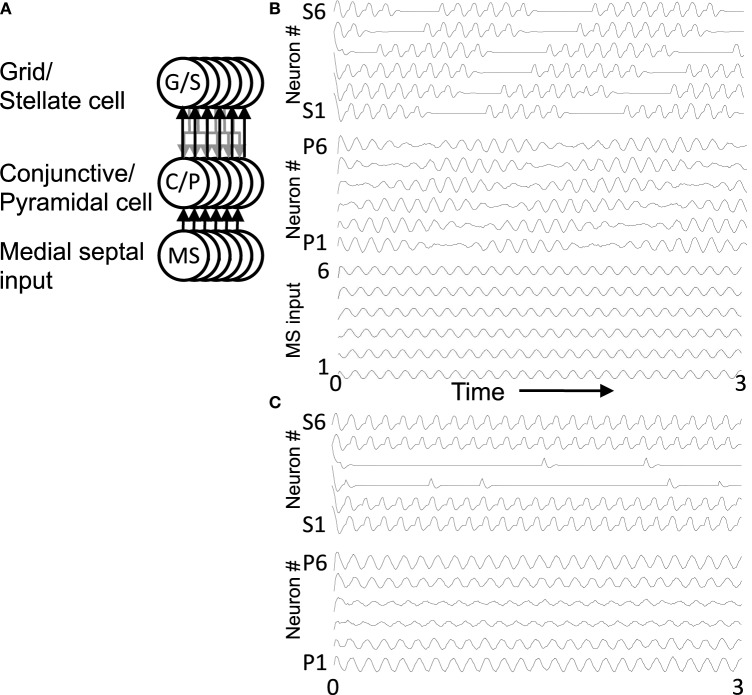
**Oscillatory activity in a full network. (A)** This circuit diagram shows a set of G/S cells, and a line of C/P cells with shared depolarizing head direction input [I_hd_ in Equation (6)]. The C/P cells receive oscillatory MS input with different phases [I_ms_ in Equation (6)]. Each C/P cell sends output to only one G/S cell in the line, but receives input from all G/S cells in the line. **(B)** The resonance frequency of G/S cells (10.2 Hz) is higher than the driving frequency from MS cells (8.1 Hz), the rebound oscillations of G/S cells activate C/P cells receiving MS input of an earlier phase, causing a shift in activity from C/P1 and G/S1 to CP/2 and GS/2 and progressively to cells of earlier and earlier phase. When the activity reaches C/P6 and G/S6, the rebound activates the cells of next earliest phase, which are C/P1 and G/S1, so the cycle repeats. **(C)** When the resonance frequency of the G/S cells is reduced to a lower value (9.7 Hz) and closer to the frequency of the MS input (8.1 Hz), then the rebound oscillations match the phase of MS input in the same population and the activity remains stationary in the same set of cells 1–2 and 5–6.

**Figure 6:**
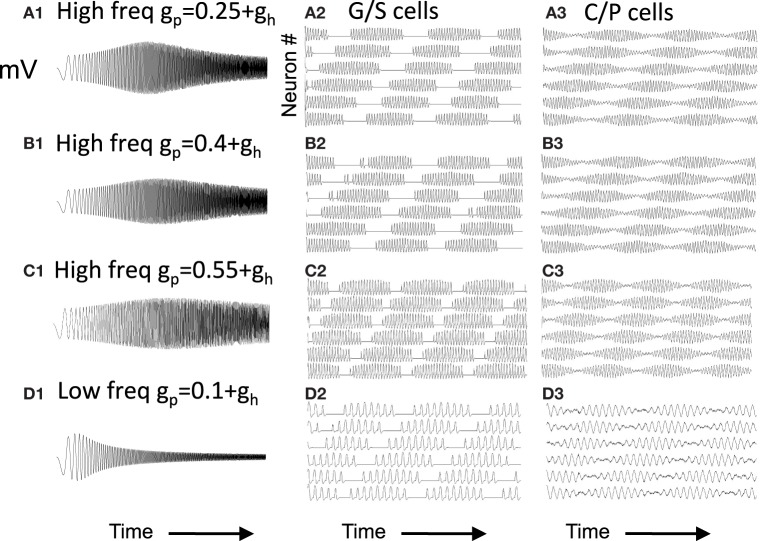
**Capacity to generate oscillatory interference patterns differs with magnitude of resonance strength.** At high frequencies **(A–C)**, high resonance strength gives patterns resembling oscillatory interference that works across a range of speeds. The parameters in **(C)** best match the experimental data in Figure 1 (*g*_*p*_ = −0.75). Beat patterns can be obtained with these parameters, but it is more difficult to maintain these beat patterns at a range of different running speeds. At lower frequencies **(D)**, lowering the network frequency of MS input still gives clear oscillatory interference.

**Figure 7:**
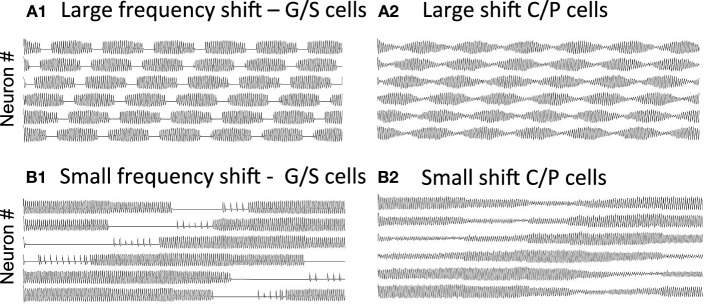
**Change in periodicity with different resonance frequencies. (A)** A larger difference in resonance frequency relative to MS input (set at 8.1 Hz) causes a faster beat frequency in the activity of grid/stellate cells **(A1)** and conjunctive/pyramidal cells **(A2)**. If running speed causes a large shift in frequency (from 9.64 to 9.89 Hz), this corresponds to narrow spacing between small grid cell firing fields. **(B)** A small difference in resonance frequency relative to MS input causes a slower beat frequency in the G/S cells **(B1)** and C/P cells **(B2)**. If running speed causes a small shift in frequency (from 9.64 to 9.66 Hz), this corresponds to wide spacing between large grid cell firing fields. The simulations in **(A,B)** used global feedback inhibition for increased stability.

The excitatory model focuses on excitatory connections of the stellate cells with excitatory pyramidal cells in layer II or in deeper layers of entorhinal cortex such as layer III and layer V, as shown in Figure 3A. In this model, the pyramidal cells are proposed to be conjunctive cells, so they are labeled with C/P in Figures 3–7, and their membrane potential is designated by the *v*_*c*_. The dynamics involved subsets of grid/stellate (G/S) cells interacting with subsets of conjunctive (C/P) cells via excitatory connections between these two classes of neurons. The activity was maintained in one location when the timing of rebound activity of grid/stellate cells matched the peak phase of activity of conjunctive/pyramidal cells driven by rhythmic input from the medial septum. This can be considered a type of attractor, in which a wave of excitatory activity caused by recurrent excitation alternates with a period of hyperpolarization that activates the h current in stellate cells and causes rebound depolarization that reactivates the same pattern of stellate activity from the previous cycle. The activity can also shift when the rebound activity in stellate cells is shifted in phase relative to the currently active pyramidal cells.

#### Inhibitory model

**Figure 8:**
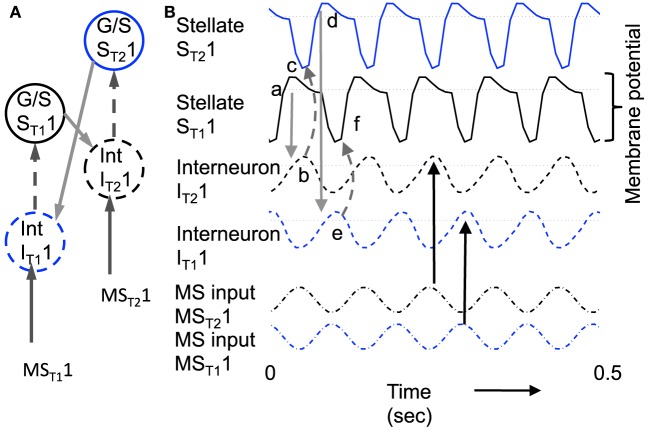
**Example of sustained oscillations with inhibitory feedback between matched pairs of neurons using stellate cells with parameters *g*_*p*_ = −0.71, *g*_*h*_ = 0.16, *m* = −1, and *o*_*h*_ = −0.35 giving resonance frequency of 10.17 Hz compared to medial septum frequency of 10 Hz. (A)** Local circuit showing excitatory connections (Solid arrows) from stellate cell with index S_T1_1 to interneuron with index I_T2_1, and from stellate cell S_T2_1 to interneuron I_T1_1, and inhibitory connections (dashed arrows) from interneuron I_T1_1 to stellate cell S_T1_1 and from interneuron I_T2_1 to stellate cell S_T2_1. **(B)** Time course of change in activation of one coupled pair of cells. **(a)** Stellate cell S_T1_1 (solid black line) starts out hyperpolarized, rebounds over the threshold shown by dotted line and sends excitatory synaptic output (gray line) to interneuron I_T2_1. **(b)** Interneuron I_T2_1 (dashed black line) is depolarized over threshold (dotted line). **(c)** Interneuron I_T2_1 sends inhibitory output (gray dashed arrow) to cause hyperpolarization in stellate cell S_T2_1 (solid line). **(d)** Stellate cell S_T2_1 rebounds from the hyperpolarization to cross threshold (dotted line), sending excitatory output to interneuron I_T1_1. **(e)** This brings interneuron I_T1_1 over threshold, sending inhibitory output (gray dashed arrow) to hyperpolarize stellate cell S_T1_1. **(f)** This causes stellate S_T1_1 to show rebound spiking to start the same cycle again.

**Figure 9:**
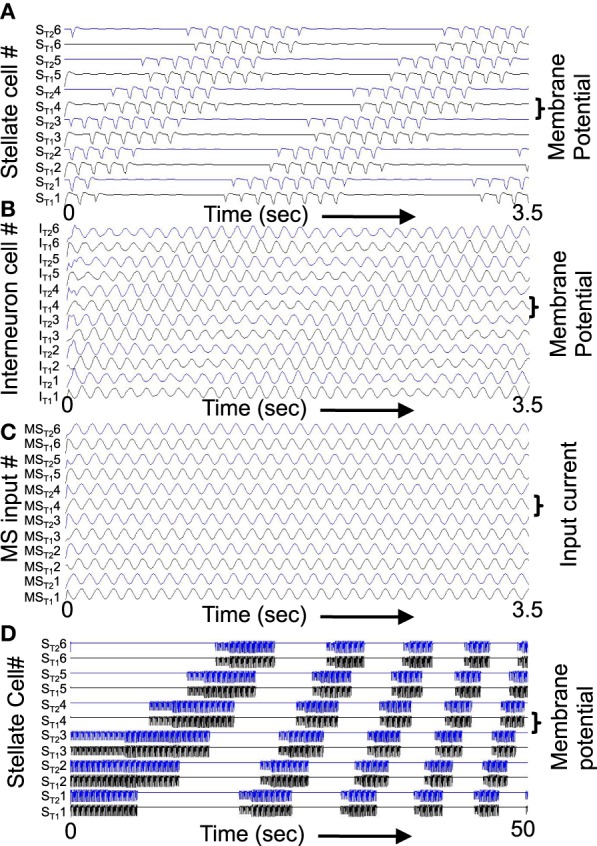
**Example of shift in population activity driven by timing of rebound from inhibition using parameters *g*_*p*_ = −0.75, *g*_*h*_ = 0.15, *m* = −1, and *o*_*h*_ = −0.35 from Figure 1. (A)** Activity of G/S neurons from S_T1_1 and S_T2_1 through S_T1_6 and S_T2_6 showing inhibitory effects of interneurons on G/S neurons visible as strong hyperpolarizing deflections. The G/S neurons with these parameters have resonance frequency of 10.2 Hz and show rebound that is slightly faster than the frequency of MS input (9.75 Hz) to interneurons. **(B)** Activity of interneurons (Int) from I_T1_1 and I_T2_1 through I_T1_6 and I_T2_6. The interneurons receive input from the G/S cells. Because the G/S cells have rebound that is slightly faster than the frequency of oscillatory MS input, the individual interneurons show a progressive shift between large amplitude oscillations when G/S input phase matches their current MS input, and periods of low amplitude when G/S input does not match their current MS input. **(C)** Oscillatory input from medial septum (MS input) showing the difference in phase of the MS input oscillations. Note that the higher indices have earlier phases (earlier peaks) than the lower indices. For example, MS_T1_2 peaks at an earlier time than MS_T1_1. **(D)** This network with resonance of 10.2 Hz can shift across a wide range of speeds by decreasing the medial septum frequency, which starts here at 10 Hz for stationary activity at *t* = 0 and decreases to 9.96 Hz at 50 s.

**Figure 10:**
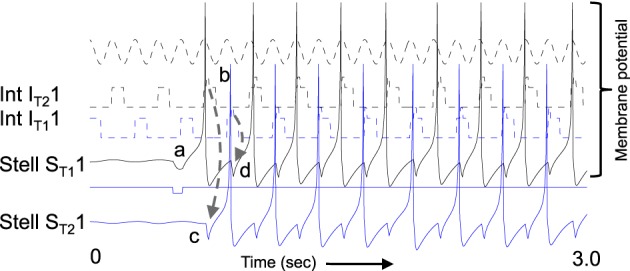
**Example of spiking model with resonance using Izhikevich neurons.** The same function shown in Figure 8 occurs for spiking neurons. A hyperpolarizing pulse (labeled with **a**) given only to G/S S_T1_1 causes a rebound spike (solid black). This brings Interneuron I_T2_1 above threshold (dashed black line) by label **b**. The interneuron sends inhibitory input to G/S S_T2_1 (label **c**). This causes a rebound spike in G/S S_T2_1 that activates interneuron I_T1_1. This causes an inhibitory potential in G/S S_T1_1 (label **d**) starting the cycle again. This process allows maintenance of spiking activity within this circuit. The spikes fall on alternating cycles of a theta rhythm oscillation shown at top (note that this theta rhythm oscillation is at double the frequency of the cycle skipping input to the interneurons).

**Figure 11:**
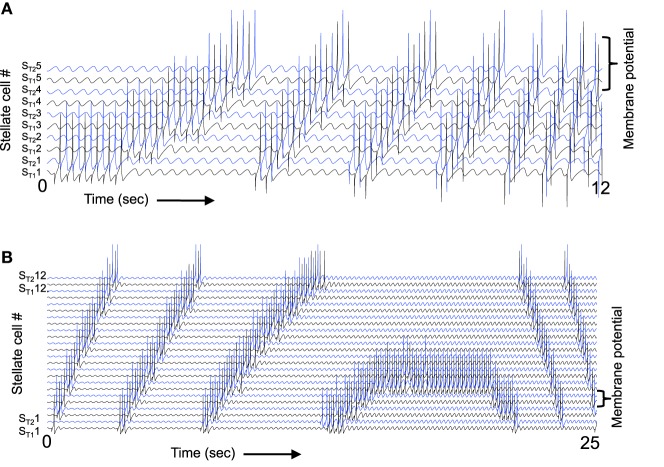
**The spiking model can respond to a wide range of different speeds. (A)** When the magnitude of feedback inhibition is progressively increased, this changes the speed of transition. Initially, the spiking activity stays stationary in stellate cells S_T1_1 and S_T2_1, but gradually as the magnitude of feedback inhibition increases the rebound spikes fall at earlier times relative to MS input. This results in a progressive shift to S_T1_2 and S_T2_2 and as the frequency difference gets larger the transition occurs more rapidly. Note that when S_T1_5 and S_T2_5 are active, the next earlier interneuron phase activates stellate cells S_T1_1 and S_T2_1, so that the sequence repeats through the line. **(B)** A continuous change in speed of transition can also be obtained by increasing the frequency of medial septal input. At the start, the frequency difference is large and the transition between neurons happens rapidly, but as the MS input frequency increases this eventually causes the rebound spiking to match the MS input resulting in stationary activity, and when MS input frequency is higher than the rebound spiking speed this causes a reversal in the direction of transitions between spiking neurons.

**Figure 12:**
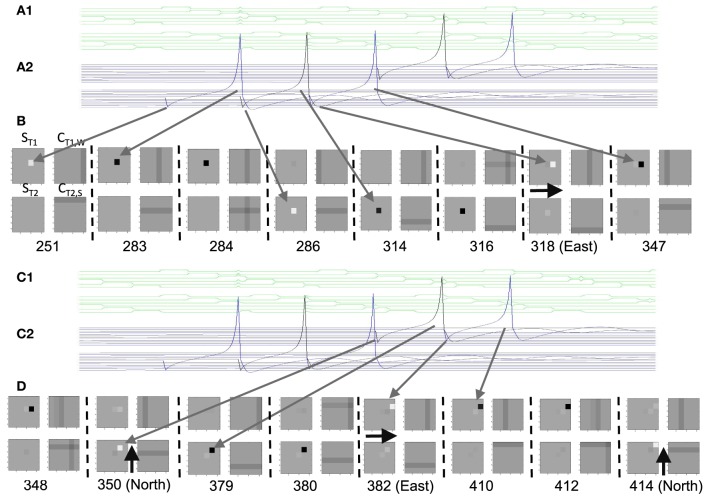
**Implementation of the inhibitory rebound model in two dimensions. (A1 and C1)** Traveling waves in the interneuron populations interact with synaptic input from the grid cell spiking (two rows of interneurons are shown). **(A2 and C2)** Spiking in two rows of stellate cells during the same period. Inhibitory input to one stellate cell at time 251 causes a rebound spike at time 283 that gives synaptic input to a column of conjunctive cells, activating the conjunctive cell at the peak of the south-moving traveling wave at step 284. This causes inhibition of the next stellate cells at step 286. **(B and D)** Two dimensional plots at specific times indicated by numbers showing stellate population S_T1_ in square 1, conjunctive cell population C_T1,W_ getting West-moving traveling wave in square 2, stellate population S_T2_ in square 3 and conjunctive cell population C_T2,S_ getting South-moving traveling wave in square 4. At time 251, the white square in S_T1_ shows an inhibitory pulse to one stellate cell. At time 283 the black square shows rebound spike in that cell. This activates a column of conjunctive cells in C_T2,S_ that converges with the row of activity due to the south-moving traveling wave at time 284. This causes inhibition (white square) in the corresponding stellate cell in S_T2_ at time 286. At time step 316, the stellate rebound spike activates a row of conjunctive cells in C_T1,W_ that converges at an earlier time with the column of cells activated by the West-moving traveling wave, activating an interneuron one step to the East. This causes inhibition in S_T1_ at a position one step to the East. Similarly, the rebound spike at time 347 converges with the South-moving traveling wave in conjunctive cells one step to the North at time 348. This causes inhibition at time 350 in a stellate cell in S_T2_ shifted on position to the North. Thus, the timing of the rebound spike relative to the traveling wave moves the spiking in different directions on different theta cycles.

**Figure 13:**
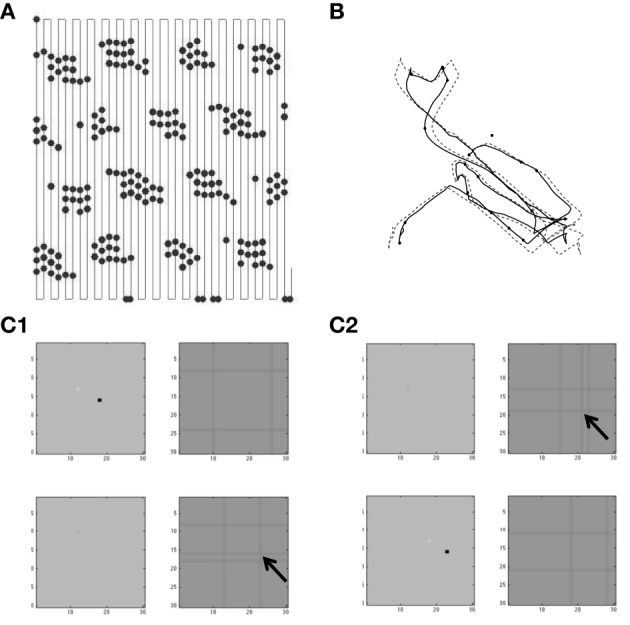
**Examples of spiking on two dimensional trajectories. (A)** Spiking of a single grid cell during a regular scanning trajectory through two dimensional space. This spiking activity has a regular pattern of locations, indicating the accuracy of spatial representation for such a regular trajectory. **(B)** Comparison of trajectory input (solid black line) with the internal spatial location based on active neurons (dashed black line). During irregular movement, the network can track the location moderately well over time. **(C)** The tracking of a trajectory with curves required larger differences in index between the neuron showing a rebound spike and the conjunctive cell responding to medial septal input waves (all four directions are combined in these plots). The first example shows stellate rebound spiking in **(C1)** (black square) that activates a conjunctive cell (arrow). This causes inhibition in a different stellate cell. The rebound spike in the same stellate cell in **(C2)** (black square) activates a different conjunctive cell (arrow). This causes inhibition in a different stellate cell to track movement in that direction.

The inhibitory model focuses on inhibitory input from interneurons with local focused connectivity to the stellate cells, as shown in Figure 3B. The simulations shown in **Figures 8–13** focus on this inhibitory input to stellate cells. The network simulations use a single population of inhibitory cells that interact with the stellate cells, but these inhibitory neurons could also receive head direction input from head direction cells (Taube et al., 1990), or could be activated by conjunctive/pyramidal cells in deep layers that respond to head direction input. Therefore, the inhibitory interneurons could also be considered to have conjunctive cell properties consistent with experimental data. The inhibitory model in **Figures 8**, **9** was simulated with continuous firing rate equations whereas the inhibitory model in **Figures 10–13** used a spiking model of layer II stellate cells in MEC developed by Izhikevich (2007) and described below.

#### Equations of the model

Simulations of the excitatory model in Figures 3A, 4–**7** and the initial inhibitory model in Figures 3B, **8**, **9** use a continuous firing rate representation of individual neurons based on the equations of resonance above. The equations representing individual neurons were replicated and combined into local circuit interactions (see Figure 3) as follows:

(4) $$dv_{s}/dt\;=\;-g_{p}v_{s}+g_{h}h_{s}-W_{si}\sum_{p}H[v_{i,p}-\eta_{p}]+I_{Sms}$$

(5) $$dh_{s}/dt\;=\;-mv_{s}-o_{h}h_{s}$$

(6) $$\begin{array}{l} \;v_{i,p}\;=\;\sum_{s}W_{is}H[v_{s}-\eta_{s}]-\mu z+I_{ms}\\ \;+\;I_{hd}\cos(\theta (t)-\theta_{p}) \end{array}$$

(7) $$\;dz/dt\;=\;-\lambda z+k\sum_{s}H[v_{s}-\theta_{s}]$$

Where *v*_*s*_ is a vector representing the different membrane potentials of the individual grid cells/stellate cells (with index *s*) within a population of grid/stellate (G/S) cells with resonance properties due to their intrinsic variables *h*_*s*_ [which represents the h current as described in Equations (1) and (2)]. *v*_*i*,*p*_ is a vector representing the different membrane potentials of a different population of cells that in the excitatory model are referred to as conjunctive/pyramidal (C/P) cells with each cell indicated by index *i*, and different sets of C/P cells with different preferred head directions θ_*p*_ designated by *p*. In the inhibitory model the activity of the conjunctive cells provide output not to the stellate cells but to interneurons with index *i* that provide one to one connectivity back to the stellate cells. The function *H*[.] is a Heaviside function, that is zero below threshold η and one for values above η. The matrix *W*_*si*_ is an identity matrix, so that each conjunctive cell or interneuron i is connected back to a single grid cell at the same position with the same index s (but the input is summed over the active conjunctive cells using the index *p*). The matrix *W*_*is*_ provides connectivity from each grid cell to all the cells within a single “line” of the conjunctive cells (that send output to interneurons) with an index of the spatial location coded by the neurons that overlaps with the position of a given grid cell. **Figures 12**, **13** showing the two-dimensional simulation in the Results section illustrate the functional effect of this connectivity pattern in which each single stellate cell contacts a “line” of conjunctive cells.

#### Medial septal input

As can be seen in Equation (6), the membrane potential of the second population *v*_*i*_ lacks effects of the *h* current and therefore lacks resonance properties. However, this population receives a driving rhythmic (sinusoidal) input from interneurons that represents neurons regulated by oscillatory input from the medial septum (MS). Experimental data suggests that the medial septum primarily contacts inhibitory interneurons in the hippocampus (Freund and Antal, 1988) and in the entorhinal cortex (Witter, personal communication). Therefore, in the excitatory model, the MS input represents input to C/P cells from local interneurons that receive oscillatory modulation from the medial septum. In the inhibitory model, the MS input in the equation represents how local interneurons gate the activity of conjunctive cells that drive the inhibitory interneurons in the model (see below).

The sinusoidal input to *v*_*i*_ regulated by medial septum in both models is represented by *I*_*ms*_ with magnitude μ = 2.8. The frequency and phase of the sinusoidal input is determined by the following equation with *x* evenly distributed between 0 and 2π so each value *x*_*i*_ = 2π*i*/*n*, where i is the integer index of the neuron in that line and n is the total number of neurons with different evenly spaced phases along the dimension of the heading direction selectivity of the conjunctive cells.

(8) $$I_{ms}=\mu \sin(2\pi ft+2\pi kx_{i})$$

This corresponds to a traveling wave where f is the medial septal frequency and k is the wave number (number of spatial cycles across the simulated population). The frequency f of medial septum was set to 8.1 Hz in Figure 4 and wave number was set to 1. This equation could be seen as a solution (Elmore and Heald, 1969) to a medial septal wave equation: $\frac{\delta^{2}v}{\delta x^{2}}={\left(\frac{f}{k}\right)}^{2}\frac{\delta^{2}v}{\delta t^{t}}$ These wave functions could occur for a wide range of heading angles as shown in equation 10 below.

The network dynamics depended on the resonant frequency determined by the parameters of the above equations relative to the frequency f of the medial septal input. When the resonant frequency was significantly different from the medial septal input, this caused a shift in activity between different neurons in the population as shown in the figures.

The speed of shift in activity could be regulated by altering the frequency of medial septal input (as one possible mechanism). As shown in the Results section, this provides a smooth influence of movement speed on the shift of activity in the network. Experimental data has shown that running speed is associated with changes in both frequency of network theta rhythm (Maurer et al., 2005) and firing rate of entorhinal neurons (O'Keefe et al., 1998; Wills et al., 2012). The even distribution of phases of medial septal input is motivated by recording of unit activity in the medial septum showing a broad distribution of different phases of firing relative to hippocampal theta rhythm (Bland, 1986; King et al., 1998).

#### Head direction input

The conjunctive cells receive depolarizing input *I*_*hd*_ based on the current head direction θ*t* of the animal relative to the preferred head direction θ_*p*_ of each population of C/P cell. This simulates the fact that conjunctive grid-by-head-direction cells respond to both the current head direction of the animal and the position of the animal relative to grid cell firing fields (Sargolini et al., 2006; Boccara et al., 2010; Brandon et al., 2011; Stensola et al., 2012). This means that changes in the relative frequency of resonance and MS input will selectively influence the relative firing of conjunctive cells for the current head direction, providing a direction component to interact with the speed component.

#### Pattern of connectivity

Most of the simulations presented here utilize arrays of neurons representing interactions along a one-dimensional trajectory through space with only two directions of movement along this trajectory, as in other recent models (Navratilova et al., 2012). However, simulations in **Figures 12**, **13** also address firing in two-dimensional environments with a full range of head direction angles as in other models (Burgess, 2008; Hasselmo and Brandon, 2012), so the description here will describe two dimensional arrays.

In simulations of the excitatory model there was a single population of G/S cells. In simulations of the inhibitory model, there were two populations of G/S cells (labeled with subscript indices “T1” and “T2”). In contrast to the G/S cells, the conjunctive cell populations are organized into head direction selective planes that consist of two-dimensional arrays of cells with dimensions x and y corresponding to position within the array (these positions differ by head direction angle relative to other arrays). In the simulations shown in **Figures 12**, **13**, there were four populations of conjunctive cells for each of the populations T1 and T2 of G/S cells. Each of these 4 populations of conjunctive cells drove a single population of inhibitory interneurons providing input back to the stellate cell populations. The conjunctive cells received phasic input from medial septum assigned relative to their *x* and *y* position and their head direction selectivity θ_*p*_ (this MS input was treated as separate populations, but would most likely contain overlap for neurons of the same phase). The total size of dimensions x and y were the same for the single population of G/S cells, the multiple populations of C/P cells and the input from medial septum (MS).

Within each plane, if the current head direction θ is close to the preferred head direction θ_*p*_ of that plane, then all the conjunctive cells receive the same input for preferred head direction *I*_*hd*_ = 1.0. The cells can be considered to be organized into lines of cells along the dimension of head direction preference, consisting of *x* different lines in each plane and *y* cells in each line. Within each line, each of the *y* C/P cells receive input of strength *W*_*is*_ = 0.5 to 0.53 from each of the full array of G/S cells in that line. This input is generated whenever the cell *v*_*s*_ is over threshold θ_*s*_ = 0.2, where the brackets [] represent the Heaviside step function which has value 0 for *v*_*s*_ < θ_*g*_ and value 1 for *v*_*s*_ > θ_*s*_. Each G/S cell *v*_*s*_ receives excitatory feedback input from the single corresponding C/P cell in population *v*_*i*_ with the same Heaviside step function computed for values above θ_*i*_ = 4. The feedback input to grid cells from conjunctive cells arrives via an identity matrix (one to one connectivity) with uniform connection strength *W*_*si*_ that varied dependent upon the resonant strength. When resonance strength was high the connections were set at 0.85 (e.g., Figure 5), whereas lower resonance strength required stronger connections up to 2.8.

#### Global inhibition

The network also included inhibition with global connectivity that helped to increase stability in the simulations. This global inhibition used a variable *z*_*i*_ that has a slow buildup similar to the time course of GABA_B_ receptor activation of potassium conductances. Each inhibitory unit was modeled as receiving input from the full population of G/S cells. Each G/S cell received uniform input of strength H from the inhibitory unit z within that head direction selectivity, so that this corresponded to uniform inhibition for a given line of cells with a slow time course.

#### Spiking neuron simulations

The simulations shown in **Figures 10–13** used the model of simplified spiking neurons developed by Izhikevich (2007). In these models, excitatory entorhinal stellate cells were simulated using the parameters (Izhikevich, 2007, p. 315) that were tuned to replicate experimental data on stellate cells from John White's lab (Burton et al., 2008). These neurons were combined with more abstract representations of conjunctive cells and inhibitory interneurons. The neurons used the following equations (Izhikevich, 2007, p. 273):

(9) $$\begin{array}{l} C\dot{v}=k(v-v_{r})(v-v_{t})-u+1\\ \;\dot{u}=a\{b(v-v_{r})-u\}\\ \;v\geq v_{peak},v\leftarrow c,u\leftarrow u+d \end{array}$$

For stellate cells, the parameters were:

*C* = 200, *v*_*r*_ = −60, *v*_*t*_ = −45, *v*_(*peak*)_ = 30, *k* = 0.75, *a* = 0.01, *b* = 15, *c* = −50, *d* = 100.

Consistent with experimental data (Couey et al., 2013), there was no direct synaptic coupling between stellate cells. Based on data on theta cycle skipping (Jeffery et al., 1995; Deshmukh et al., 2010; Brandon et al., 2013), the stellate cells in the simulations in **Figures 10–13** were split into two populations S_T1_ and S_T2_ that fired on alternating cycles of the theta rhythm. Synaptic coupling between the stellate cells and conjunctive cells used step functions that were active for one time step after a spike, in order to preserve the detailed timing of spikes relative to the phase of medial septal traveling wave input to conjunctive cells. The medial septal input was structured as square wave inputs that did not overlap in the depolarized phase, so only a limited number of conjunctive cells at a given time or phase were sufficiently depolarized to be able to respond to the synaptic input from the spiking stellate cells. This was done separately for two groups of four different populations of conjunctive cells representing different directions (e.g., C_T1,W_ represents a conjunctive population associated with stellate cell population T1 with a west-moving traveling wave). These groups of four directions of conjunctive cells drove single interneuron populations that provided inhibitory feedback to the stellate cell populations. The spiking model had the interesting characteristic that running speed could be represented by the strength of feedback inhibition, because the speed of rebound spiking depended upon the magnitude of hyperpolarizing pulses. This is discussed in more detail in the Results section.

### Model with wide range of wave directions

**Figure 14:**
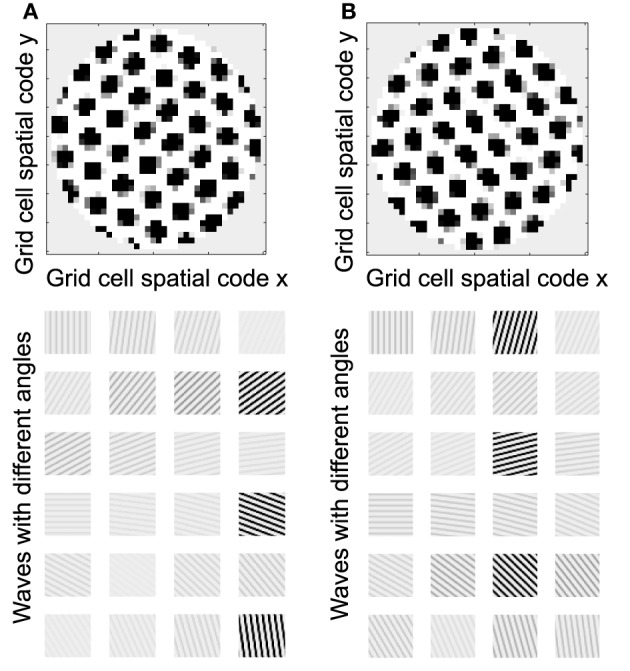
**Feedback interaction between transverse traveling waves with 48 different heading angles.** (**A** top) The feedback interaction described in Equation (11) results in a grid cell pattern across the population of simulated neurons. (**A** bottom) The feedback involves grid cells causing feedback regulation of the amplitude and phase of input traveling waves. The bottom row shows the magnitude of traveling waves at 24 out of the 48 heading angles. Each orientation in the population corresponds to different maximal feedback activation of waves with different heading angles (but always at intervals of about 60°). With 48 heading angles, these come at intervals of 7.5°, so that 60° corresponds to every 8th heading angle wave being maximally active. **(B)** Example of a simulations with different initial conditions shows that activity across the population of grid cells settles into different predominant orientations (top) that correspond to different maximal feedback activation of traveling waves (bottom) with different heading angles (but always at intervals of about 60°).

**Figure 15:**
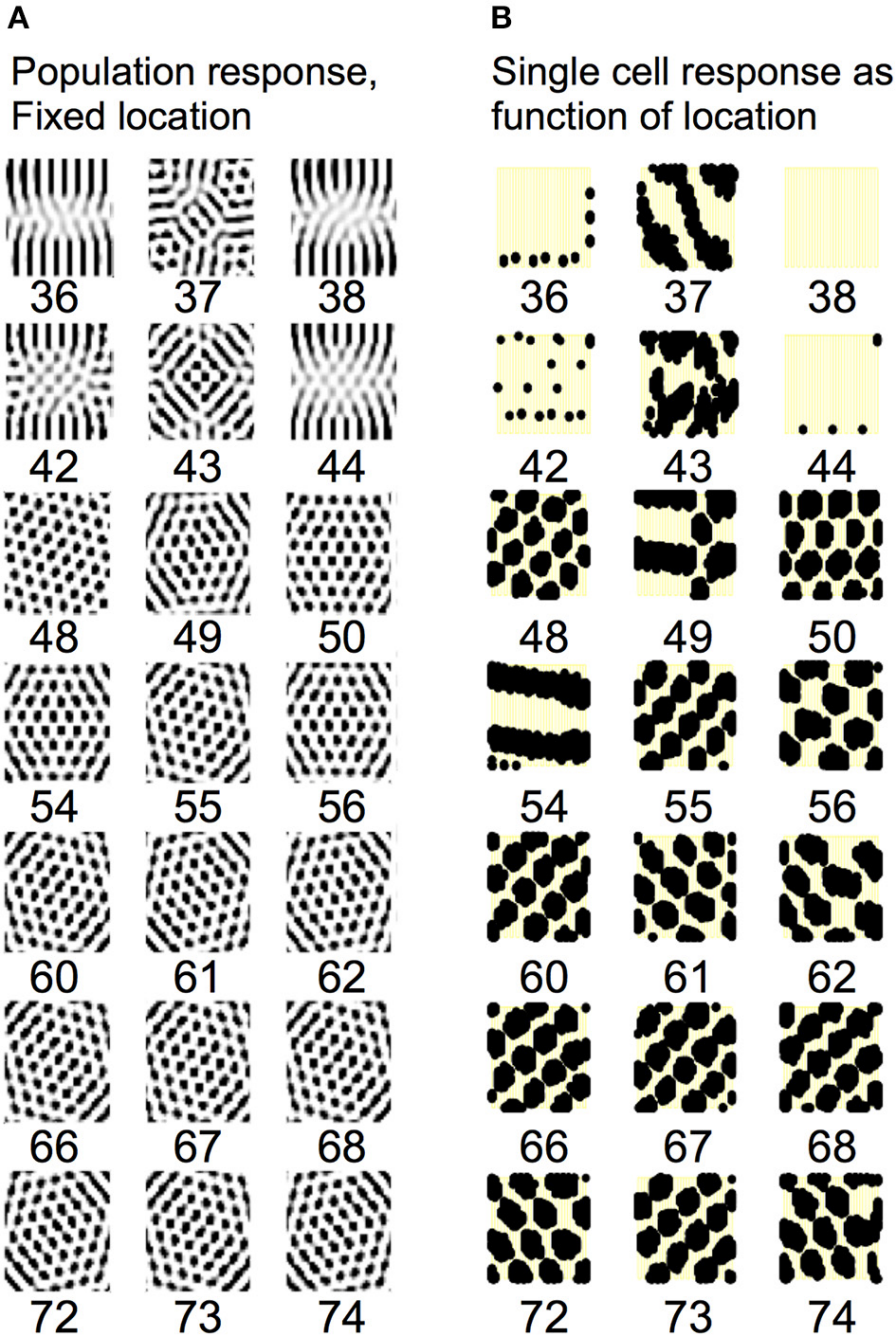
**The network functions well with large numbers of heading angles. (A)** For 48 or more heading angles (starting on third row) the network consistently generates hexagonal patterns of firing across the population. But the hexagonal pattern does not consistently appear for smaller numbers of heading angles (e.g., 36 or 42). **(B)** The firing activity of single neurons during movement is most reliable for 55 or more heading angles (right side). There is a lack of consistent firing for lower numbers of heading angles (e.g., 36 or 42) but the pattern is more consistent for larger numbers of heading angles.

Understanding the role of resonance in entorhinal cortex neurons requires understanding the response of entorhinal neurons in terms of oscillations in time. Understanding how resonance could influence the representation of different spatial phases by grid cells requires the use of oscillations of different phases coding different spatial locations. The need for both temporal phase and spatial phase indicates that the network might be modeled in terms of waves of activity. Note that the spatial dimension of these waves is not the anatomical position of cells within the cortex, as in many previous models (Ermentrout and McLeod, 1993; Coombes, 2005; Meijer and Coombes, 2013), but in terms of the location within the environment coded by individual cells. The simulations in **Figures 14**, **15** focused on showing how the feedback interaction with populations of traveling waves with many different directions could result in a dominant influence of waves with a limited range of directions.

As an initial example of how interacting waves could generate the grid cell firing pattern within a population, **Figures 14**, **15** show a model in which waves with multiple different directions of propagation have feedback interaction with a population of entorhinal cells to generate a grid cell firing pattern within the population and within individual cells. The wave in this model corresponds to the models by Blair and Zhang showing that input from ring attractors representing oscillatory input from theta cells in the medial septum or thalamus can generate grid cell firing patterns (Blair et al., 2008; Welday et al., 2011). In the Blair papers, the oscillating rings are referred to as ring attractors and use one dimensional rings of neurons. In the equations below, the ring attractors are described as generating two-dimensional transverse traveling waves that propagate across the plane of grid cells representing an environment. The representation of animal velocity in these models depends upon shifts in the pattern of interference between oscillations, so the model falls in the category of oscillatory interference models developed by Burgess et al. (2007), Burgess (2008).

Modelers that use attractor dynamics with circularly symmetric synaptic feedback to generate grid cells have criticized oscillatory interference models for assuming that the velocity controlled oscillators must be constrained to have preferred heading angles at intervals of 60°. However, the simulations presented in this section show that an oscillatory interference model can generate hexagonal grid cell patterns with velocity controlled oscillators driven by inputs with a wide range of arbitrary input angles. This was shown using a variant of the oscillatory interference model presented in an earlier publication in which multiple directions of two-dimensional transverse traveling waves interact to generate grid cells (Hasselmo and Brandon, 2012).

Several equations were used to describe the model in that previous paper (Hasselmo and Brandon, 2012), but the function of that previously published model can be greatly clarified here in a different mathematical description that allows summary with a single equation:

(10) $$\begin{array}{l} g_{xy}(t)\;=\;\tau g_{xy}(t-1)+\sum_{x^{\prime }y^{\prime }}\sum_{\phi_{i}}\cos\left(\omega t-\vec{\kappa }(\phi_{i})\cdot (\vec{r_{xy}}-\vec{r_{x^{\prime }y^{\prime }}})\right)\\ \;{\left(g_{x^{\prime }y^{\prime }}(t-1)-\Omega \right)}_{+}/\text{ max}\left(g_{x^{\prime }y^{\prime }}(t-1)\right) \end{array}$$

Where *g*_*xy*_(*t*) represents the activity at time *t* of an array of grid cells that code a set of locations *x*,*y* in the two-dimensional spatial environment being explored by the rat. In Equation (10), the grid cell activity is updated by input from a large array of transverse traveling waves (referred to as heading angle planes in the previous paper) that provided input to the two-dimensional array of grid cells. As in the previous model, each location in Equation (10) is represented by a full set of traveling waves coding a range of heading directions ϕ (each with a different index *i*) in the rat environment. The angle ϕ of propagation of each traveling wave is represented by the direction of a unit vector κ, related to the direction angle ϕ and wave number k as follows $\vec{\kappa }$ (ϕ_*i*_) = [*k*cos ϕ_*i*_ *k*sin ϕ_*i*_]. This is a standard representation for the direction of two-dimensional transverse, traveling waves (Elmore and Heald, 1969). Thus, each transverse, traveling wave propagates across the plane of spatial locations in a different direction ϕ. The traveling waves have temporal frequency ω.

The equation shows how the input to each grid cell *g*_*xy*_(*t*) is influenced by the sum of traveling waves arising from the full array of locations *x*′*y*′ with the full set of angles ϕ at each location. The spatial phase of each of these waves is computed by the dot product of the vector κ(ϕ_*i*_) with the vector computed from the difference between the full array of location vectors *r*_*xy*_ = [*x* *y*] and the vector representing the central location of that traveling wave *r*_*x*′*y*′_ = [*x*′ *y*′]. This results in the spatial phases $\vec{\kappa }$ (ϕ_*i*_) ($\vec{r}$_*xy*_ − $\vec{r}$_*x*′*y*′_).

Each of the arrays of traveling waves with spatial phases corresponding to the location *x*′*y*′ with the set of angles ϕ had a magnitude in proportion to the level of previous grid cell activity *g*_*x*′*y*′_(*t* − 1) coding each of the locations *x*′*y*′ at time *t*-1. The grid cell activity was put through a threshold linear function with threshold Ω. Equation (10) shows that in the model in the previous paper (Hasselmo and Brandon, 2012), the set of traveling waves corresponding to each location *x*′*y*′ are first summed over all of the angles ϕ_*i*_, giving concentric circles around the location *x*′*y*′. Subsequently, the grid cell activity *g*_*xy*_(*t*) at the current time step *t* was determined by summing over all of the spatial phases corresponding to summation over all locations *x*′*y*′ (plus the current activity scaled by τ = 0.3).

In simulations of that previous model, a set of two-dimensional traveling waves was generated with a range of 24 different heading angles evenly distributed over 360° and a wave number of 4 across the full environment being coded by a rat (Hasselmo and Brandon, 2012). The full set of 24 traveling waves was generated for each coded location, and summation generated a circularly symmetric standing wave pattern. The magnitude of the standing wave was then regulated by the activity of each individual grid cell on the previous time step. Thus, the previous model performed summation over spatial locations subsequent to summation over heading angles. The interaction of the grid cells and the traveling waves resulted in population activity settling into a pattern of grid cell activity as shown in multiple figures in that paper (Hasselmo and Brandon, 2012). One important problem with this model is that the circularly symmetric standing waves generated by summation across angles would have different spatial locations of peak firing on different half cycles. This was avoided by the use of two populations with π phase difference, and the use of only half the cycle for each. The mechanism for this constraint was not simulated but could be achieved by cross-inhibition between the two populations.

The computation of this model can be made more efficient by changing the order of summation to speed the computation process and to describe the input as a smaller array of traveling waves. The new mathematical description of the previous model allows simplification by changing the order of the summation and by adding a different mechanism for movement. This allows the previous paper to be extended by showing simulations for much larger range of interacting angles and full two-dimensional trajectories that were not previously tested.

Thus, the summation can be performed as follows:

(11) $$\begin{array}{l} g_{xy}(t)=\tau g_{xy}(t-1)+\sum_{\phi_{i}}\sum_{x^{\prime }y^{\prime }}\cos(\omega t-\vec{\kappa }(\phi_{i})\cdot \left(\vec{r_{xy}}-\vec{r_{x^{\prime }y^{\prime }}}\right)\\ \;-H(\phi_{i})\Delta \vec{x}(t)){\left(g_{x^{\prime }y^{\prime }}(t-1)-\Omega \right)}_{+}/\text{ max}\left(g_{x^{\prime }y^{\prime }}(t-1)\right). \end{array}$$

In Equation (11), for the traveling waves coding a particular heading direction, the waves are summed over all the phases corresponding to the grid cells coding all locations *x*′*y*′. In addition, the computation can be further speeded if instead of explicitly computing and summing the full set of traveling waves, the circular mean phase and mean amplitude are computed across the full array of waves representing the angle ϕ_*i*_ at every location *x*′*y*′. This different order of summation first generates the amplitude and phase of a single traveling wave for each heading direction angle ϕ_*i*_, and then sums these waves across the full set of direction angles. The waves generated before summation across angles can represent the input from a single cyclical traveling wave for each angle, which could be generated by a mechanism such as the previously proposed ring attractors (Welday et al., 2011), instead of requiring a full array of ring attractors for coding of each location.

As noted above, after computing the phase of each traveling wave across the population, the grid cell activity *g(t)* at the current time step is determined by the summation over all of the angles ϕ_*i*_ of the different traveling wave input. This allows the input from transverse traveling waves with a large range of preferred directions (heading angles) to generate an interference pattern in the grid cell population that settles into a hexagonal grid cell firing pattern, and ensures that all the waves have the correct relative phase when location is updated, allowing a shift in the driving force of velocity when the animal turns. Note that this model still requires the use of two sets of waves offset by phase π to avoid the shift in coded location. A gap of π/8 between the waves was used in simulations shown here, τ was set to 0.5, and the normalization factor was squared to reduce overall amplitude. Note that the direct feedback excitation used here and in Hasselmo and Brandon (2012) differs from the modification of synaptic input used in models of the self-organization of directional input (Mhatre et al., 2012) or place cell input (Kropff and Treves, 2008; Si et al., 2012).

Equation (11) includes an additional element *H*(ϕ_*i*_)Δ*x*(*t*) that represents the effect of the current velocity Δ*x*(*t*) of the animal on the phase of individual waves. The effect of velocity on each wave is scaled by one row of the matrix *H*(ϕ_*i*_) which computes the cosine and sine for each heading angle ϕ_*i*_ with *H*(ϕ_*i*_) = [cos ϕ_*i*_ sin ϕ_*i*_]. Because the network updates the phase of all waves, the location update also works with narrower functions more similar to the tuning of real head direction cells, such as rectified cubes*H*(ϕ_*i*_) = [cos^3^_+_ ϕ_*i*_ sin^3^_+_ ϕ_*i*_]. Note that equations 10 and 11 are using cosine functions for waves rather than simulating them in a neural circuit with the rebound properties of neurons, but these traveling waves can be considered a potential solution to wave equations for this network.

## Results

The system summarized by Equations (4)–(8) provides dynamics that allow progressive shifts of activity in grid/stellate (G/S) cells and conjunctive/pyramidal (C/P) cells. Figure 4A shows the conditions without feedback between the C/P and G/S cells. The input from the medial septum (MS) shown in Figure 4A drives oscillations that remain subthreshold in a single C/P neuron *v*_*i*_ (Figure 4A row MS). Starting from a depolarized potential, the resonance properties of the G/S cell causes a single rebound depolarization that brings it above threshold. In the absence of excitatory connections, the C/P cells do not come over threshold.

Figure 4B shows the effect of bidirectional excitatory connections (arrows in circuit diagram). The G/S cell starts with depolarized initial conditions, but the addition of excitatory connections allows this activity to drive the C/P cell over threshold and the neurons drive each other. In contrast to fixed-point attractors, the activity does not persist in a static manner. The activity decreases because the MS input to the C/P cell decreases in amplitude and because the depolarization shuts off the h current in the G/S cell, allowing the G/S cell to drop below threshold and subsequently later show a rebound depolarization that brings it above threshold again. The rebound depolarization over threshold causes further output that drives the C/P cells over their threshold (gray dashed line). The C/P cell excitatory feedback drives the G/S cell to higher activity. As the MS input to the C/P cell decreases and the h current shuts off, both the C/P cell and the G/S cell fall below threshold again, but the G/S cell shows rebound depolarization on the next cycle, driving the C/P cell over threshold and pushing the G/S cell to the same depolarized level. In this manner, the two cells continue to excite each other and maintain a repeating oscillatory interaction throughout this example. This does not require continuous activity as in traditional fixed-point attractors. Instead, oscillatory activity is maintained with low firing rates across theta cycles by the use of intrinsic rebound depolarization. This resembles the rebound spiking used in Navratilova et al. (2012), but uses subthreshold dynamics that allow simulation of subthreshold resonance properties.

### Movement of activity between multiple stellate-pyramidal pairs

A shift in the oscillatory activity can occur when multiple C/P neurons with different phases of input from medial septum interact with multiple G/S neurons, as shown in Figures 4C, 5A. This allows a shift in the phase of maximal activity that is proportional to the difference in resonance frequency in the G/S cells relative to the MS input frequency to the C/P cells. In this example, the rebound depolarization occurs early relative to the MS input oscillations to C/P. This corresponds to the phase shift caused by the currents underlying resonance.

Figure 4C shows how this difference between resonance frequency and MS input causes a shift in phase for a single G/S neuron and relative to a single C/P neuron when both are part of a network shown on the left. The black lines in the circuit diagram show the neurons illustrated in this example, and the gray lines in the circuit diagram show other neurons present in the simulation whose traces are not shown. In this network, the C/P neurons each receive the same frequency of MS input, but with a different phase. The G/S neurons each receive input from their corresponding single C/P neuron (black upward arrow), but they send output to the full line of C/P neurons at all phases (downward line that forks into many branches).

The G/S neuron in Figure 4C starts out above threshold, and interacts with the C/P neuron shown here, but on each cycle its rebound depolarization occurs a bit earlier, causing it to send stronger output to another C/P cell that receives MS input with an earlier phase. Because the C/P cell with earlier phase of MS input is closer to threshold, this causes it to generate greater output, which does not go to the G/S cell shown here, but goes to the G/S cell associated with the earlier phase C/P cell. Thus, the activity transitions to a C/P cell with an earlier phase of MS input, and the corresponding G/S cell. As the first G/S cell shown here goes out of phase with the MS input to its corresponding C/P cell, the amplitude of oscillation in both of these neurons decreases over the first 5–6 cycles.

The activity transitions to different pairs with different phases until it cycles through the full network and starts to activate the C/P and G/C cells illustrated here to start an increase in the envelope of oscillations (that appears as a beat pattern). This beat pattern looks like an oscillatory interference model, but the model presented here uses intrinsic oscillations (modeled by differential equations) rather than fixed sinusoidal functions for the interaction. In these differential equations, the beat frequency depends upon the difference in resonance frequency of the G/S cell and the frequency of MS input to the C/P cell. This links the properties of oscillatory interference models directly to the resonance properties of layer II stellate cells in entorhinal cortex.

The shift in phase of the G/S cells relative to the MS input resembles theta phase precession of MEC grid cells as shown in the oscillatory interference model (Burgess, 2008) and in experimental data from the Moser laboratory on a linear track (Hafting et al., 2008). Theta phase precession also appears in two-dimensional data based on the distance since a rat enters a grid cell firing field (Climer et al., 2013). In the figure, precession also occurs in the C/P cell consistent with precession appearing in some conjunctive cells in deeper layers (Climer et al., 2013) including layer V pyramidal cells (Misuzeki et al., 2009). However, many layer III conjunctive cells do not show precession (Hafting et al., 2008).

Figure 5 shows the transition of activity in a full population of six G/S cells and six C/P cells receiving input from six MS cells with different phases. A difference in resonance frequency relative to the MS input frequency is present in all the G/S cells in Figure 5B. The parameters are *g*_*p*_ = −0.49, *g*_*h*_ = 0.24, *m* = −1, and *o*_*h*_ = −0.35, resulting in resonance frequency *f* = 10.2 Hz relative to MS input frequency of 8.1 Hz. The full array of G/S cells all have the same resonance frequency. Initial conditions start activity in cells G/S1 to G/S3. These cells show rebound depolarization that is early relative to their coupled C/P cells, so that their output activates the C/P cell with an earlier phase (C/P4) and this activates the associated G/S cell (G/S4). As this process continues, it progressively activates C/P and G/S pairs with earlier phase (e.g., C/P5 and G/S5), causing a shift in the amplitude of activity to neurons with earlier phase.

When the activity reaches the earliest phase pair C/P6 with G/S6 at the top of each line, it starts to activate the first pair in the line which is the next earliest phase. In this manner, the cyclical nature of phases allows activity to be propagated repeatedly through the network, providing a rationale for the repeating firing fields of grid cells that requires a twisted torus connectivity in fixed point attractor models. Note that this process can work in the complete absence of inhibition, because the shift in phase of activity allows C/P neurons to drop below firing threshold, and terminates the end of the activity distribution across the population. However, addition of inhibition in the model makes the mechanism more robust across a wider range of parameters.

With lower resonance frequency that is closer to the MS input frequency (or with a different MS input frequency), the network can maintain a stable activity state in a single location, as shown in Figure 5C. The parameters are all the same except that parameter *g*_*h*_ is set to 0.206 in Figure 5C so that resonance of G/S cells is 9.7 Hz. Note that even though there is still a difference in frequency, the network dynamics end up being stationary. In this example, cells 1, 2, 5, and 6 maintain activity for an extended time period (beyond the period shown), and the other pairs remain inactive. Note that C/P3 and C/P4 still get input from the active cells, but input shows destructive interference with MS input, and therefore G/S3 and G/S4 are primarily inactive (flat lines) except for occasional bumps of activity.

The shift in activity across the population of neurons shown in Figure 5B is considered to represent the movement of the animal in one direction. If the difference in frequency in Figure 5 represents the speed of the rat as it moves in a straight line in one direction, the activity would transition between different elements of this population of neurons to represent the shift in location. This resembles how oscillatory interference models generate bands of constructive interference due to interactions of velocity controlled oscillators (Burgess et al., 2007; Burgess, 2008). In this simulation, the relative phase inputs from medial septum provide a mechanism for competition between neurons. The grid cells could interact with different conjunctive cells oriented in different directions, as in the model using conjunctive cells with six directions of tuning described by Burgess (2008).

As shown in Figure 2, the magnitude of resonance strength corresponds to the magnitude of rebound depolarization. Because of this, the magnitude of resonance strength corresponds to the ease of simulating oscillatory interference. However, as shown in Figure 6, the oscillatory interference beat patterns can be generated with a range of different parameters even corresponding to the magnitude of resonance strength that resembles the experimental data.

Note that the capacity for generating beat patterns does not depend upon the intrinsic resonance frequency of the neuron. The low resonance frequency shown in Figure 6D can generate a beat pattern similar to the beat pattern generated in Figures 6A,B, as long as the network frequency is reduced to interact with the resonance frequency. The spatial periodicity of the beat pattern does not depend upon the baseline resonance frequency or the frequency of input from the medial septum, but instead depends upon the magnitude of the difference between the stellate cell resonance frequency and the medial septum input frequency. Thus, the difference in resonance frequency found in bats and rats when recording from layer II neurons in MEC does not mean that resonance frequency does not contribute in some manner to the generation of grid cell spatial periodicity. However, the framework shown in Figure 6D would require lower frequency input from medial septum in the bat. The possible presence of such lower frequencies has not yet been tested in published results from bats.

The model can account for different spacing of grid cell firing fields dependent on the mapping of running speed to resonance frequency. In simulations, the change in resonance frequency can be achieved by altering the parameter *g*_*h*_ in the first set of equations describing the continuous firing rate model. However, one difficulty is that the change in speed differs for the speed relative to different directions of movement. This is less of an issue if the conjunctive cells represent all directions of movement at high resolution, but it is a factor if there are a smaller number of directions represented. As an alternative, changes in speed can be coded by changes in the frequency of medial septal (MS) input, as shown in **Figures 9**, **11B**, or possibly in the magnitude of the synaptic feedback inhibition within the circuit as shown in **Figure 11A**.

As shown in Figure 7A, if there is a large shift in resonance frequency with running speed, then the large difference between resonance frequency and MS input causes a rapid beat frequency. This would cause narrow spacing between smaller grid cell firing fields, consistent with the recording of higher resonance frequency found in dorsal MEC (Giocomo et al., 2007; Giocomo and Hasselmo, 2008a,b; Boehlen et al., 2010; Pastoll et al., 2012a) where grid cell firing fields are smaller and closer together (Hafting et al., 2005; Sargolini et al., 2006). In contrast, if there is a small shift in resonance frequency with running speed, the smaller difference from MS input frequency causes a slower beat frequency. This could cause wider spacing between larger grid cell firing fields, consistent with the lower resonance frequencies found in ventral MEC (Giocomo et al., 2007; Boehlen et al., 2010; Pastoll et al., 2012a) where grid cell firing fields are larger and spaced further apart (Hafting et al., 2005; Sargolini et al., 2006). The simulations in Figure 7 used global feedback inhibition to enhance stability. This involved a buildup of inhibition proportional to *k* = 0.05 for each time step that a stellate was above threshold η, with a decay of λ = 0.01. This influenced the network with inhibitory magnitude μ = −0.0928.

Changing resonance frequency with running speed causes a shift in the beat frequency. However, shifts in resonance frequency with changes in speed do not give very stable transitions. For example, in simulations not shown, the running speed alters the parameter *g*_*h*_ systematically, and MS input frequency is 8.1 Hz, using G/S activity with *g*_*p*_ = −0.4585 or *g*_*p*_ = −0.75 during a smooth increase in *g*_*h*_ directly scaled to time (simulation not shown). The transitions are moderately consistent using *g*_*h*_ = 0.2064 (*f* = 9.64 Hz) and going to *g*_*h*_ = 0.2364 (10.03 Hz). However, the transitions become rather random starting with *g*_*h*_ = 0.15 (10.22 Hz) and increasing to *g*_*h*_ = 0.155 (10.28 Hz). Even with a smaller range of frequencies, the network breaks down with very small changes in the parameter *g*_*h*_. Thus, the speed modulation is unstable in the excitatory model with parameters that match the experimental data in Figure 1.

Because the beat patterns depend upon the difference in resonance frequency and MS input frequency, the network models in Figure 7 can generate a full range of beat frequencies that correspond to the spatial periodicity of a grid cell model. These one-dimensional spatial periodicities could potentially be combined together to generate a two-dimensional grid cell pattern in the same manner as described for sinusoid functions in Burgess (2008). As in the oscillatory interference model, the two-dimensional spatial periodicity in the model depends upon the scaling of resonance frequency to running velocity. Thus, this model could use elements of oscillatory dynamics to link the intrinsic property of resonance frequency in stellate cells to the features of grid cell responses, allowing a model to simulate the experimental data on larger spacing between firing fields in more ventral entorhinal cortex (Hafting et al., 2005) based on the lower resonance frequency of ventral stellate cells (Giocomo et al., 2007).

### Simulation of movement with inhibitory feedback

The excitatory connectivity used in Figures 1–7 requires a biphasic rebound response of grid/stellate cells. The G/S cells are driven to strong firing by excitatory feedback from C/P cells. This shuts off the h current and causes the cells to hyperpolarize below threshold. This activates the h current, causing an afterdepolarization that brings the cells over threshold again. As shown in Figure 2, this rebound is more robust for a certain range of modeling parameters.

In contrast, inhibitory input can cause a monophasic rebound, as the cell can directly respond to inhibitory hyperpolarization by a rebound excitation. This could allow more effective rebound and network function with resonance parameters that do not work for excitatory feedback. Therefore, the model was tested with inhibitory feedback mediated by connections from inhibitory interneurons that connect selectively to the associated grid/stellate cells as shown in Figure 3. The connection from stellates to interneurons could occur via conjunctive/pyramidal cells to provide the head direction sensitivity.

Figure 8 shows the function of a model using inhibitory feedback in a matched pair of cells as shown in Figure 3C. Because immediate feedback inhibition would counteract the spiking activity of a cell, the inhibitory feedback cannot be immediate, but instead uses the delayed rebound effect in a different cell pair to cause delayed feedback inhibition. As shown in Figure 8A, this mechanism requires coupling each G/S and interneuron pair (designated with subscript T1 in the figure, indicating one theta cycle) with a second G/S and interneuron pair (designated subscript T2 in the figure, indicating a second theta cycle). The pairs are defined by one to one coupling of the interneuron (e.g., Int_T1_1) to the G/S cell in the same pair (e.g., G/S_T1_1), whereas crossed connections link the G/S cell (e.g., G/S _T1_1) to the opposite interneuron (e.g., Int _T2_1). This circuitry results in a pattern of spiking on alternate theta cycles that has support from recent unit recording data in our laboratory (Brandon et al., 2013). This model also predicts that coupled pairs of neurons firing on opposite cycles should share the location of their firing fields.

The dynamics of this circuit are shown in Figure 8B. In this figure, Grid/Stellate cell S_T1_1 starts out hyperpolarized causing it to rebound above firing threshold (dotted line) in step a of Figure 8. The excitatory synaptic output from G/S cell S_T1_1 possibly via a C/P cell depolarizes interneuron I_T2_1 to bring it above threshold (step b). The output from inhibitory interneuron I_T2_1 then causes hyperpolarization in the G/S cell S_T2_1 (step c) causing a rebound depolarization (step d). G/S cell S_T2_1 sends excitatory output (possibly via a C/P cell) to interneuron I_T1_1. This brings interneuron I_T1_1 over threshold (step e) causing hyperpolarization in G/S cell S_T1_1 (step f). The rebound of G/S cell S_T1_1 from hyperpolarization starts the entire cycle again, so that the oscillatory interaction is sustained within the circuit. This provides a form of maintenance of oscillatory activity that maintains activity using the resonance properties mediated by h current in stellate cells to provide the rebound from hyperpolarization. Note that both the G/S cells and the interneurons in the circuit show firing on opposite cycles of an underlying network rhythmicity, consistent with data on theta cycle skipping in the MEC (Jeffery et al., 1995; Deshmukh et al., 2010; Brandon et al., 2013) and input from cells in the medial septum that show theta cycle skipping (King et al., 1998; Varga et al., 2008). The maintenance of activity due to feedback inhibition resembles previous mathematical models of activity maintained by rebound spiking using different currents (Wang and Rinzel, 1993; McCarthy and Kopell, 2012).

These inhibitory dynamics provide the same capabilities for transition between attractor states as the excitatory connections in the earlier figures, as shown in Figure 9. Figure 9 shows the neural activity across a set of 6 different pairs of grid/stellate cells (both the S_T1_1 and S_T2_1 grid/stellate cells are shown) and across 6 different associated interneurons (again both the I_T1_1 and I_T2_1 interneurons). The shift in activity is caused by increasing the parameter *g*_*h*_ in the model so that the resonance property has frequency 10.2 Hz and therefore the rebound from inhibition is faster relative to the frequency of MS input (9.75 Hz) to the pyramidal cells, causing a progressive shift in activity. Figure 9D shows that a progressive shift in the MS input frequency changes the speed of transition between different populations.

### Simulation using rebound spiking with inhibitory feedback

The rebound from inhibition that works in the abstract model also works in a network model using a more biologically realistic single neuron model of spiking and resonance properties in MEC developed by Izhikevich (2007). The single neuron model simulates the subthreshold resonance and oscillatory dynamics of medial entorhinal stellate cells, and shows resonance similar to the abstract model. Figure 10 shows that this model can function similar to the abstract model. Activity is initially induced by a hyperpolarizing input to stellate cell S_T1_1 (label a) that causes a rebound spike. The stellate cell spike activates interneuron I_T2_1 (label b) which causes an inhibitory hyperpolarizing potential in stellate cell S_T2_1 (label c). This hyperpolarizing input causes a rebound spike in stellate cell S_T2_1 after a delay. This rebound spike activates interneuron I_T1_1, which sends inhibitory input to stellate cell S_T1_1 (label d). This causes a rebound spike in the first stellate cell S_T1_1 to restart the cycle. In this manner, the circuit maintains spiking activity with different neurons firing on alternate cycles of the network theta rhythm oscillation. This is consistent with data on theta cycle skipping shown in unit recordings from awake, behaving animals (Jeffery et al., 1995; Deshmukh et al., 2010; Brandon et al., 2013).

Figure 11A illustrates that the spiking model with inhibitory feedback can hold a stable state for a period of time and also transition between neurons at a wide range of different frequencies. In the start of this simulation, stellate cell S_T1_1 maintains its activity due to the mechanism shown in Figure 10. Because the timing of the rebound spikes matches the timing of the MS input to the interneurons. As the simulation progresses in Figure 11A, the magnitude of feedback inhibition from the interneurons gradually increases proportional to each time step. As the strength of feedback inhibition increases, the time of the rebound spike occurs at earlier phases relative to the medial septum input to interneurons. This causes a progressive shift in the spiking activity of different interneurons, which activate spiking in different stellate cells. Thus, the activity spreads from stellate cells S_T1_1 and S_T2_1 to the interneurons I_T1_2 and I_T2_2 to the stellate cells S_T1_2 and S_T2_2, and then on to S_T1_3 and S_T2_3 and eventually to S_T1_5 and S_T2_5. When the rebound spiking occurs earlier than S_T1_5 and S_T2_5, the next preceding phase is interneurons I_T1_1 and I_T2_1, so the cycle repeats itself through the network. This network model has multiple different interneurons receiving square wave rather than a sinusoid input from the medial septum, with no overlap between the phasic input to different interneurons. Square wave input helped maintain stability by preventing the excess spread of activity in the network, but stability should be feasible using sinusoidal input in a larger scale network. Excess activity was prevented in a different one-dimensional network model with a single interneuron and sinusoidal input to stellate cells (Hasselmo, 2013). That model has not yet been extended to two dimensions, but could be used to obtain grid cell activity using a small number of interneurons that fire at all spatial locations with different phases relative to sinusoidal input to stellate cells.

As the magnitude of feedback inhibition increases continuously in Figure 11A it causes a more rapid transition between different spiking neurons, corresponding to a higher speed of movement through the environment. This simulation demonstrates that a spiking network can show a shift in the location of activity due to rebound from inhibition as shown by the more abstract model in Figure 9. The speed of the transition through the network can be altered by changing the magnitude of feedback inhibition.

Alternately, the network activity can be also shifted at different speeds by changing the relative frequency of medial septal input, as shown in Figure 11B. If the frequency of medial septal input wave starts out low (left), this gives a period of the medial septal input wave that is slower than the interval of rebound spiking and the network activity shifts rapidly. As the medial septal input frequency is increased, this results in a smaller difference between the oscillation period and the rebound spiking interval, causing a slowing of the shift. The shift eventually becomes static (Figure 11B center) when the period matches the rebound spiking interval. Subsequently, as the period of the medial septal input waves becomes shorter than the rebound spiking interval, this causes movement in the reverse direction. Thus, the speed of movement in the network can also be regulated by the temporal frequency of medial septal traveling wave input. As can be seen in Figure 11B, this gives a continuous transition between different frequencies, as it changes the duration of the phases when each neuron can be activated. This predicts that some septal neurons should show speed modulation of firing frequency, and specifically might show decreases with increased speed. Preliminary data indicates speed modulation of septal neurons. The frequency only needs to shift by 3.5 Hz relative to baseline to cover the wide range of speeds shown in Figure 11B.

### Simulations using two-dimensional trajectories

The mechanism of shift between neurons based on rebound spiking has been expanded to a representation of two dimensional movement. The example shown in Figure 12, shows how the framework from Figures 10, 11 can work to cause shifts in cell firing in two different movement directions on different theta cycles. Stellate cell rebound spiking shown in Figure 12A2 interacts with traveling wave input from medial septum spreading through the conjunctive populations to activate interneuron populations in Figure 12A1. This results in movement of activity within the stellate cell population in different directions during different cycles.

The same activity is shown as part of two-dimensional plots of population activity in Figure 12B, with white squares showing inhibition in a single stellate cell, and black squares showing spiking in a single stellate cell. Section A1 of the figure shows the traveling wave propagating sequentially as a square wave through two columns of conjunctive cells. The traveling wave interacts with synaptic input from the grid cell that activates a single column of conjunctive cell spiking to bring one conjunctive cell over threshold. Section A2 shows spiking in two rows of stellate cells.

Note that the simulations used for Figures 12, 13 used four different conjunctive cell populations getting distinct directional traveling waves for each stellate cell population. In the figures only the population getting the West-moving traveling wave is shown as C_T1,W_ and only the population getting the South-moving traveling wave is shown as C_T2,S_. These conjunctive cell populations then contact single interneuron populations that provide inhibitory input to each stellate cell population. In this manner, the simulation represents the effect of four different excitatory conjunctive cell populations getting the traveling wave input that thereby gate the activity arriving in two interneuron populations (so that there would be a much larger population of excitatory conjunctive cells than interneurons).

At time 251 in Figure 12, inhibitory input to one stellate cell visible in both A2 and B S_T1_ causes a rebound spike at time 283 visible as a spike in the membrane potential in A2 and a black square in B S_T1_. This activates a column of conjunctive cells in C_T2,S_ that converges with the row of activity due to the South-moving traveling wave at time 284. The convergence causes a conjunctive cell spike that activates inhibitory interneurons to cause inhibition (white square) in the corresponding stellate cell in S_T2_ at time 286. At time step 316, the stellate rebound spike activates a row of conjunctive cells in C_T1,W_ that converges at an earlier time with the column of cells activated by the West-moving traveling wave, activating a conjunctive cell one step to the East. This activates inhibitory neurons to cause inhibition in S_T1_ at a position one step to the East. Similarly, the rebound spike at time 347 converges with the South-moving traveling wave in conjunctive cells (in C_T2,S_) one step to the North at time 348. This causes inhibition at time 350 in a stellate cell in S_T2_ shifted one position to the North.

The process continues with the rebound spike at 379 converging with the traveling wave in the conjunctive cells one position to the East in 380, resulting in inhibition of a stellate cell one step to the East at time 382. This causes rebound spiking at time 410 that converges with the conjunctive cell traveling wave at time 412 and causes inhibition of a stellate cell one step to the North at time 414. Thus, the timing of the rebound spike relative to the traveling wave moves the spiking in different directions on different theta cycles. But the specific two different directions are only visible when comparing the stellate cell populations. The net effect in individual stellate cells is to cause a diagonal movement. The angle of this movement can be altered by changing the relative frequency of the traveling wave or the speed of rebound spiking for the populations representing different directions.

The example in Figure 12 shows the mechanism for the two-dimensional model to correctly shift the locus of spiking activity in proportion to movement in the two-dimensional environment. As shown in Figure 13A, this mechanism is reasonably accurate for a trajectory that scans across two-dimensional space with long segments in the north-south direction and only short movements in the eastward direction. Note that the internal model shifts in proportion to movement in the east-west direction and in response to the other movement relative to a reference vector at 60° in the southwest to northeast direction corresponding to multiplying each location (*x,y*) by the operator: $H=\left[\begin{array}{c} \cos\theta_{1}\;\sin\theta_{1}\\ \cos\theta_{2}\;\sin\theta_{2} \end{array}\right]$ where θ_1_ = 0 and θ_2_ = π/3. This means that a 90° angle difference in the actual trajectory is transformed to a 60° angle in the internal representation, resulting in periodicity with the topology of a twisted torus, that causes a hexagonal periodicity. The accurate shift in spiking activity within the population can be seen by the plot of a single neuron from the center of the population that spikes in a regular hexagonal array of locations (Figure 13A).

For certain parameters, the network can track more complex two-dimensional trajectories with frequent movement and with changes in speed. The accuracy of multiple directions of movement requires that rebound spiking be able to interact with the wave of medial septal input at large intervals. For example, a rebound spike in a stellate cell with index (1,1) might occur at the time of a medial septal input wave that causes an conjunctive cell to spike at location (1.5) and the next rebound spike may activate a conjunctive cell at location (4,5). The larger shift in neuron index in each direction is necessary to maintain accurate angles of movement. With this property, the network can track a trajectory with continuous curves, as shown in Figure 13B. In this figure, the actual trajectory is compared with an internal representation derived from the index of the stellate cell firing a spike on each time step. Shifting of the parameters allowed a reasonable match between the actual trajectory and the internal population activity in some cases (as shown in Figure 13B). As shown in Figure 13C, the accurate movements at a range of angles required larger steps in the index of conjunctive cells activated by the rebound spike of stellate cells combined with the medial septal input to conjunctive cells. Note that the examples in Figure 13C show very narrow waves of medial septal input, but it should be feasible to obtain similar function with smoother input waves from medial septum. This would allow future simulations to extend beyond single neuron spiking within the two-dimensional population, to obtain a delimited two-dimensional region of spiking activity that shifts with running velocity.

### Feedback interactions select small number of traveling waves

As noted in the Methods section, the interaction of waves of activity was also explored in a more abstract model with feedback interactions between grid cells and traveling wave inputs. The goal of this model was to understand how a large population of grid cells could interact with a large number of different head direction inputs. This more abstract model does not yet include rebound spiking, but could ultimately be combined with the model of rebound spiking. This more abstract model is similar to the model published in Hasselmo and Brandon (2012) but uses a simpler mathematical representation that is clearer to describe and more efficient to implement, allowing much more extensive analysis of different numbers of heading angles.

Figure 14 shows the grid cell firing pattern resulting when a large number of different traveling waves are combined. The feedback interaction causes traveling waves that are not at 60° intervals to be decreased in magnitude by the dynamics of the circuit, as shown in the figure. This allows effective generation of grid cells in the model. Different random initial conditions can result in different orientations of the hexagonal pattern across the population corresponding to different maximal activation of the heading angle waves providing input.

Figure 15 shows that shifting the frequency of individual traveling waves can allow the activity to be moved around the network. Note that in this simulation, traveling waves can interact to generate a stationary grid pattern, and then be shifted if the relative frequency of traveling waves is shifted (Blair et al., 2013). Figure 15 shows that a simple trajectory through the environment that can generate a two-dimensional grid cell firing pattern. Figure 15 also shows that the hexagonal pattern of neuronal activity is only consistently obtained for large numbers of heading angles above 48 in Figure 15A and above 55 in Figure 15B.

## Discussion

The models presented in Figures 1–13 uses simulated neurons that replicate the resonance properties of stellate cells in MEC shown in extensive experimental studies of these neurons (Haas and White, 2002; Erchova et al., 2004; Giocomo et al., 2007; Engel et al., 2008; Fernandez and White, 2008; Giocomo and Hasselmo, 2009; Boehlen et al., 2010; Heys et al., 2010). The resonance allows rebound depolarization to mediate rhythmic interactions with conjunctive/pyramidal cells or interneurons receiving theta rhythmic input from the medial septum. The rhythmic interactions allow maintenance of oscillatory activity in subsets of neurons within the population. When the intrinsic resonance frequency of the stellate cells is increased relative to the medial septum input frequency, or the medial septum input frequency is decreased, this causes rebound depolarization to cause a shift in the activity state between different sets of neurons within the model. The movement along a two-dimensional trajectory can be obtained by skipping between shifts in different directions during alternating cycles of theta rhythm oscillations.

The patterns of increases and decreases of activity in different subsets of neurons resembles the beat patterns in the oscillatory interference model of grid cells (Burgess et al., 2005, 2007; Blair et al., 2008; Burgess, 2008; Hasselmo, 2008) and related models (Nadasdy, 2009, 2010). But the use of coupled differential equations in Figures 1–9 allows a systematic change in amplitude of oscillations, rather than just changing the phase and frequency as in previous implementations of the oscillatory interference model. This provides a framework for describing how differences in resonance frequency at different dorsal to ventral positions (Giocomo et al., 2007; Giocomo and Hasselmo, 2009; Boehlen et al., 2010; Pastoll et al., 2012a) can cause the difference in spacing of grid cell firing fields along the dorsal to ventral axis (Hafting et al., 2005; Sargolini et al., 2006; Stensola et al., 2012). This model also provides a potential framework for linking resonance at low frequencies in bat entorhinal cortex (Heys et al., 2013) to the generation of grid cells in bats (Yartsev et al., 2011; Barry et al., 2012a). As long as resonance strength is sufficiently strong, the model shows that the beat patterns providing spatial periodicity can be generated with resonance at lower frequencies similar to data recorded from the entorhinal cortex of two species of bat (Heys et al., 2013).

Note that this model links resonance frequency to the deterministic timing of rebound depolarizations, but does not require the noise-driven subthreshold oscillations of entorhinal stellate cells (Dickson et al., 2000). This is an important advantage for this model, as subthreshold membrane potential oscillations do not have reliable time course (Zilli et al., 2009) and cannot show independent frequency and phase within a neuron (Remme et al., 2010). The use of rebound depolarizations in this model resembles the mechanism of maintaining activity patterns in a model of short-term memory (Lisman and Idiart, 1995) and to encode sequences for modeling theta phase precession (Jensen and Lisman, 1996). The same mechanism was also used to maintain an attractor in a previous paper (Navratilova et al., 2012). The mechanism of attractor maintenance is similar in focusing on rebound depolarization, but that previous paper triggered the intrinsic mechanisms with a spike in an integrate and fire model (Navratilova et al., 2012), rather than due to subthreshold rebound from inhibition. Also, the attractors were more directly shifted by a velocity input, rather than being indirectly shifted by the effects of velocity on the relative phase of interaction of resonant cells with cells receiving MS input. In the model presented here, the loss of MS input prevents grid cell periodicity, as supported by data showing loss of spatial periodicity of grid cells with inactivation of the medial septum (Brandon et al., 2011; Koenig et al., 2011). The previous model does not depend upon MS input, and must also attribute periodic boundary conditions to patterns of synaptic connectivity rather than the cyclical nature of the phase of oscillatory input from the medial septum as used here.

Another previous model generated grid cell firing patterns based on waves of activity moving across the entorhinal cortical sheet (Nadasdy, 2009, 2010) to allow coding of sensory input by spike phase. Decoding of this phase signal relative to repeated waves in two dimensions resulted in hexagonal periodicity. That model predicted a hexagonal distribution of grid cell coding across the cortical surface, as supported by a recent paper (Ray et al., 2014). That model did not use intrinsic rebound spiking properties or traveling waves oriented with multiple head directions.

This current model addresses the increase in spacing observed in mice with the forebrain restricted knockout of the HCN1 subunit of the h current (Giocomo et al., 2011) which alters the parameters of the h current so that cells do not show resonance at theta frequency, but instead show resonance at lower frequencies (Giocomo and Hasselmo, 2009). Here this difference was simulated by altering the parameter *g*_*h*_ in Equation (1) and showing that resonance could still mediate grid cell periodicity. If the lower frequency resonance shows smaller frequency shifts relative to velocity, this would cause broader spacing between grid cell firing fields. Thus, the model is supported by experimental data showing larger spacing between firing fields in more ventral entorhinal cortex (Hafting et al., 2005) and lower resonance frequency of ventral stellate cells (Giocomo et al., 2007), and by data showing that knockout of the HCN1 subunit of the h current causes lower resonance frequency (Giocomo and Hasselmo, 2009) and wider spacing of firing fields (Giocomo et al., 2011). The model predicts a total loss of grid cell periodicity with total loss of rebound spiking if h current were completely removed by loss of both HCN1 and HCN2 subunits. The two-dimensional model properties (Figures 12, 13) are supported by recent data showing lack of shared head direction tuning for neurons that fire asynchronously on alternating cycles of theta rhythm (Brandon et al., 2013), and predicts that there should be coupled pairs of asynchronous neurons that fire on opposite cycles but share the same location of their firing fields, while coding different directions of movement and changing their firing pattern on different cycles. In addition to support from data showing loss of grid cell periodicity with medial septum inactivation (Brandon et al., 2011), the model is supported by data (Welday et al., 2011) showing cosine-tuned directional modulation of septal neurons that could be part of ring attractors generating traveling waves. The model also predicts that septal neurons should show speed modulation of firing frequency, and that sets of septal cells should show shared orientation tuning but different temporal theta phase offsets consistent with traveling waves. Note that the model is consistent with evidence of resonance in neocortical and hippocampal circuits (Stark et al., 2013).

The model presented here also provides a potential framework for addressing the effects of acetylcholine on the resonance properties of neurons (Heys et al., 2010; Barry et al., 2012c) and the theorized role of this effect in underlying the experimentally demonstrated change in spacing of grid cells in novel environments (Barry et al., 2012b). A change in acetylcholine levels could cause a shift in overall speed modulation (Newman et al., 2013, 2014), or could be directly involved in modulating intrinsic resonance frequency relative to running speed. Acetylcholine levels have been shown to change in running vs. stationary rats (Mizuno et al., 1991; Marrosu et al., 1995) and have been shown to change the resonance frequency of medial entorhinal neurons (Heys et al., 2010; Heys and Hasselmo, 2012). Alternately, resonance frequency could be shifted by systematic changes in the resonance frequency of neurons due to different levels of depolarization with running speed. Experimental data shows that the resonance frequency decreases with depolarization in MEC (Shay et al., 2012).

The model presented here has a potential advantage over attractor models in that it can maintain activity with low firing rates at theta frequency. Traditional attractor models use slow excitatory synaptic currents to maintain activity over long delay intervals between theta cycles. For example, the models by Pastoll et al. (2012b) uses NMDA currents with time constants of 100 ms, which is longer than the time constant of excitatory synaptic potentials demonstrated in the entorhinal cortex (Garden et al., 2008).

The prominence of head direction sensitivity in driving MEC neurons (i.e., both head direction cells and conjunctive cells) suggests the importance of sensory input for updating grid cells, requiring constant monitoring of the orientation of sensory input to the head. The spherical coordinates of each sensory stimulus relative to the animal could influence the phase and amplitude of specific traveling waves, and this could be included in the summation of traveling waves described in the model presented in Figures 14, 15.

In this framework, in addition to integrating body movement, the system may involve learning a link between sensory features and particular phases of traveling waves. This influence on waves could include visual features, but also input from a rodent's whiskers, and auditory input. The influence on waves could involve an interaction of head direction with the relative bilateral sensory input in the two eyes or two whisker systems. A rotational shift in sensory angle in the same direction on both sides of the head corresponds to pure head movement without body movement and should not alter the phase of waves. But movement with the head direction held sideways relative to movement should shift the phase of waves lateral to head direction. If sensory flow is in opposite directions for the two eyes for a given head direction during forward movement then this should shift the phase of waves parallel to head direction. In addition to addressing the predominance of head direction input, a framework driven primarily by sensory input could address the change in firing patterns such as the expansion or compression of grid field spacing with changes in environment size (Barry et al., 2007; Stensola et al., 2012) and the linearization of grid cell firing in the hairpin maze (Derdikman et al., 2009). To allow sufficient influence of input on the attractor state it may be necessary to change the relative strength of input vs. feedback on different phases of theta rhythm (Hasselmo et al., 2002).

In simulating grid cell firing properties, a modeler does not need to choose between oscillatory interference and attractor dynamics, as these are not incompatible models, as shown by Bush and Burgess (2014). The model presented here uses elements of oscillatory interference, but the effects of excitatory or inhibitory feedback could be interpreted as resulting in dynamical attractors within the model. The model shows that resonance frequency indicates the time course of rebound depolarization that allows generation of beat patterns by interaction of resonance frequency with the frequency of input from the medial septum. The model shows that with sufficient resonance strength, beat patterns can be generated with a range of different resonance frequencies. Figures 12, 13 show that the one-dimensional firing patterns generated by rebound oscillations can be combined into two-dimensional grid cell firing patterns as proposed in previous models of grid cell firing patterns (Burgess, 2008; Navratilova et al., 2012).

### Conflict of interest statement

The authors declare that the research was conducted in the absence of any commercial or financial relationships that could be construed as a potential conflict of interest.
